# Comparative metabolomics of MCF-7 breast cancer cells using different extraction solvents assessed by mass spectroscopy

**DOI:** 10.1038/s41598-019-49509-y

**Published:** 2019-09-11

**Authors:** Mohammad H. Semreen, Hasan Y. Alniss, Stefan R. Grgic, Raafat A. El-Awady, Ahmed H. Almehdi, Muath K. Mousa, Rifat A. Hamoudi

**Affiliations:** 1 College of Pharmacy, University of Sharjah, P.O. Box 27272, Sharjah, United Arab Emirates; 2 Sharjah Institute for Medical Research, University of Sharjah, P.O. Box 27272, Sharjah, United Arab Emirates; 3 Faculty of Medicine, Department of Pharmacy, University of Novi Sad, P.O. Box 21001, Novi Sad, Serbia; 4 Department of Chemistry, College of Sciences, University of Sharjah, P.O. Box 27272, Sharjah, United Arab Emirates; 5 Research Institute of Science and Engineering, University of Sharjah, P.O. Box 27272, Sharjah, United Arab Emirates; 6 College of Medicine, University of Sharjah, P.O. Box 27272, Sharjah, United Arab Emirates; 7 Division of Surgery and Interventional Science, University College London, London, United Kingdom

**Keywords:** Medical research, Molecular biology

## Abstract

Metabolic profiling of cancer cells can play a vital role in revealing the molecular bases of cancer development and progression. In this study, gas chromatography coupled with mass spectrometry (GC-MS) was employed for the determination of signatures found in ER+/PR+ breast cancer cells derived from MCF-7 using different extraction solvents including: A, formic acid in water; B, ammonium hydroxide in water; C, ethyl acetate; D, methanol: water (1:1, v/v); and E, acetonitrile: water (1:1, v/v). The greatest extraction rate and diversity of metabolites occurs with extraction solvents A and E. Extraction solvent D showed moderate extraction efficiency, whereas extraction solvent B and C showed inferior metabolite diversity. Metabolite set enrichment analysis (MSEA) results showed energy production pathways to be key in MCF-7 cell lines. This study showed that mass spectrometry could identify key metabolites associated with cancers. The highest enriched pathways were related to energy production as well as Warburg effect pathways, which may shed light on how energy metabolism has been hijacked to encourage tumour progression and eventually metastasis in breast cancer.

## Introduction

Metabolic profiling of cancer cells and biomarkers identifications has drawn the attention of many researchers to understand the complexity and diversity of cancer cell biology. Metabolomics has helped researchers to reveal specific biomarkers for many diseases, and that might improve both personalized therapies and clinical outcomes after primary diagnosis^1^. Breast cancer is the second leading cause of death and the most common cancer in women globally^2^ and while there are many screening methods such as mammography^3^, Breast Self-Examination, and Clinical Breast Examination. However, high throughput and advanced molecular platforms are essential for the detection and characterization of novel biomarkers for disease-associated metabolic disorders and improved early diagnostic methods are desirable. Breast cancer is highly heterogeneous and consists of at least 10 different disease sub-types^4^. Breast cancer involve many different cellular pathways including metabolic and hypoxia related pathways such as the Warburg effect related pathways^5^. Warburg effect is when cancer cells keep a high glycolytic rate even in aerobic conditions indicating that glycolysis is vital for cancer survival and progression^6^.

Metabolomics is a promising analytical tool which is described as a large scale investigation of small molecules (metabolites) in a biological sample that are characterized using mass spectrometry or other suitable biophysical techniques. The metabolome is defined as the metabolites and their interactions with their targets within a biological system^7^. Due to the complexity of metabolome, there are three main analytical techniques currently applied in metabolomics: mass spectrometry (LC–MS)^8^, gas chromatography–mass spectrometry (GC–MS)^9,10^, and nuclear magnetic resonance (NMR)^11–13^. The huge advancement in the applications of these modern analytical techniques allow the analysis of large metabolomics data at different molecular levels, including the organism, organ, tissue, cell and organelle levels. Such data can provide critical information about biomolecular function and the biochemical relationships of metabolites, which might lead to a better understanding of the biological system under investigation.

Gas chromatography–mass spectrometry (GC-MS) is the most commonly used and standardized technique in metabolomics, with more than 50 years of established protocols for metabolite characterization of amino acids^14^, hydroxyl acids^15^, fatty acids^16^, catecholamines^17^, sugars^18^, hormones^19^, and many other metabolic intermediates.

In the last four decades, mass spectra have been accumulated and stored in libraries under standard conditions of 70 eV electron ionization energy^20–22^, More importantly, tremendous efforts have been made to computationally match mass spectra with experimental data to help in the identification of compounded via their unique fragmentation pattern^23–25^.

In comparison, GC-MS/MS libraries are more comprehensive and updated compared to LC-MS/MS libraries. Furthermore, peak picking in GC is associated with true mass spectral deconvolution, which summarize all fragment ions into a purified mass spectrum, whereas in LC-MS methods, the MS and data dependent MS-MS fragmentations are used separately. This was facilitated by the availability of an automated mass spectral deconvolution software (AMDIS), which has been successfully used in GC-MS for metabolomics studies since 1998^26–28^. GC-MS is more selective, robust, rapid and easier to identify metabolites due to the available of ready-made libraries. Therefore, it can be said that GC-mass spectrometry provides higher resolution for metabolite separation.

Tumour cells have altered metabolism, therefore their metabolic mapping is considered a promising tool in cancer diagnostics^29^. Different pathways are subjected to alteration within the cancer cells during the development of the disease, which might affect the metabolism of cancer cells compared to the normal cells^30–32^. Among the affected pathways is glycolysis, and its end product pyruvate, represents a key intermediate in several metabolic processes linked to energy production such as carbohydrate and fatty acid, which are important components to cell proliferation^33,34^. However, little has been reported on the quantitation of metabolic intermediates involved in energy metabolism (such as metabolites of glycolysis, Krebs cycle and oxidative phosphorylation) in cultured breast cancer and normal cells^35,36^. This is mainly due to the fact that most metabolites involved in energy metabolism are characterized by high polarity, low volatility, and thus poor detectability.

In this study, we explore the optimal metabolite extraction protocol from MCF-7 breast cancer cells^37^ and the subsequent metabolic profiling utilizing gas chromatography coupled with tandem mass spectrometry (MS). GC-MS analysis of cultured MCF-7 tumour cells provided detailed information about their metabolic profiling.

## Results

### Assessment of metabolic extraction

**Figure 1:**
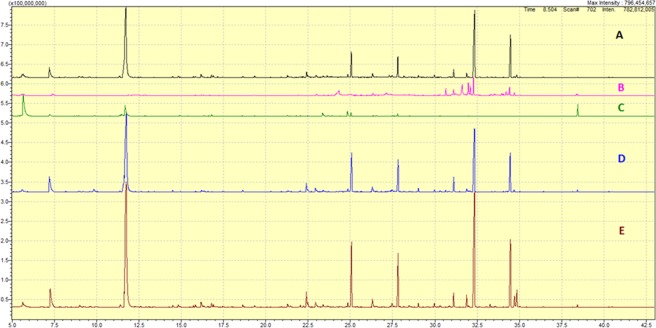
GC-MS chromatograms of the extracted cells generated using: (**A**) 0.2% formic acid in water; (**B**) 0.2% ammonium hydroxide in water; (**C**) ethyl acetate; (**D**) methanol/water (1:1, v/v); and (**E**) acetonitrile/water (1:1, v/v).

The GC/MS chromatograms of the extracted cells using the five extraction solutions under investigation are shown in Fig. 1, the greatest extraction rate and diversity of metabolites is noticed when employing 0.2% formic acid and acetonitrile: water mixture, whereas 0.2% ammonium hydroxide and ethyl acetate showed inferior metabolite diversity, whereas methanol: water showed moderate extraction efficiency.

A total of 395 metabolites were detected and characterized in the extracts under investigation, as shown in Fig. 1 and Table 1.

**Table 1:** Summary of metabolic extraction yield of the investigated extraction solutions. The data was generated from the analysis of triplicate samples.

Metabolic Extract	Extraction Solvent	Detected metabolites	Identified metabolites	# of metabolites with RSD < 15%
A	0.2% formic acid in water (F.A)	111	103	60
B	0.2% ammonium hydroxide in water (NH_4_OH)	41	36	16
C	ethyl acetate (E.A)	76	66	15
D	methanol/water (MeOH: H_2_O)	93	83	41
E	acetonitrile/water (MeCN: H_2_O)	120	107	69

Supplementary Tables (S1–S5) has details of the solvent metabolite extraction data.

Among the aqueous-based extraction solutions A (acidified with 0.2% formic acid) and B (0.2% ammonium hydroxide), A showed higher extraction efficiency in terms of metabolites detection and identification with overall coverage of 111 against 41. According to the data presented in Table 1, extraction solution E (acetonitrile/water), A (acidified with 0.2% formic acid) and D (methanol/water) demonstrated greatest superiority in overall metabolome coverage, with number of metabolites identified 107, 103 and 83 respectively. The identification cutoff was based on the signal: noise ratio of the peaks. The reproducibility of the extraction efficiency was evaluated by calculating the RSD values of the identified metabolites. The results showed that the highest reproducibility was achieved using solvents E and A (RSD < 15% in 69 and 60 of the metabolites identified in E and A, respectively), while solvent D provided moderate reproducibility (RSD < 15% in 41 metabolites), and low reproducibility was observed with solvents B and C (RSD < 15% in 16 and 15 metabolites for B and C respectively).

Variety of fatty acids, hydroxyl acids, saccharides and their metabolites (glycolysis, Krebs cycle, polyol pathway) were detected by employing most of the solvents, excluding 0.2% ammonium hydroxide and ethyl acetate. However, optimal chromatographic conditions (resolution and peak intensity) were achieved by employing extraction solution A. Ribitol was found to be efficiently extracted with solution C (ethyl acetate) and D. Surprisingly, non-conventional metabolites (Stigmasterol and its metabolite) were detected and identified in the MCF-7 cells.

Five metabolites were identified and detected in all extraction solvents used in this study at different concentration levels. This includes Inositol sugar, which is a polar and neutral molecule; this may explain its existence in aqueous and organic solvents. Moreover, two fatty acids (Palmitic and stearic acids) were found in all solvents used in this study at different concertation levels and this is mainly related to the coexistence of two states (ionized and non-ionized) of these fatty acids, which is pH dependent.

Two molecules were detected (Octasiloxane-hexadecamethyl and Heptamethyl-3,3-bis(trimethylsiloxy)tetrasiloxane) in all extraction solvents, however, these species were generated as a by-product of the derivatization process, which is an essential step to enhance the lipophilicity of metabolites to improve peak symmetry and metabolite GC detection.

**Figure 2:**
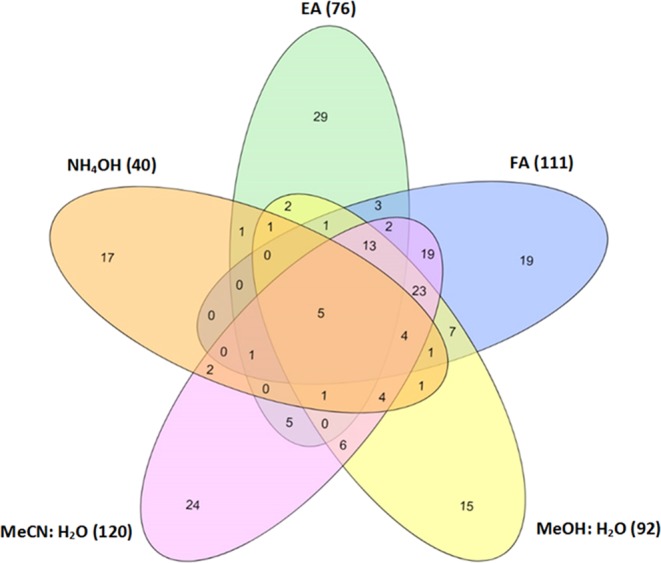
A Venn diagram showing the detected metabolites in ammonium hydroxide (NH_4_OH), ethyl acetate (EA), Formic acid (FA), methanol: water (MeOH: H_2_O) and acetonitrile: water (MeCN: H_2_O) extracts.

Venn diagram representation in Fig. 2, confirmed that the highest overlap of metabolite identification was achieved based on the polarity of the extraction solvents. In this case, the acetonitrile:water extracted 120 metabolites and formic acid (0.2%) extracted 111 metabolites compared to the other solvent extractions.

### Association between different extraction methods on MCF-7 breast cancer cells

**Figure 3:**
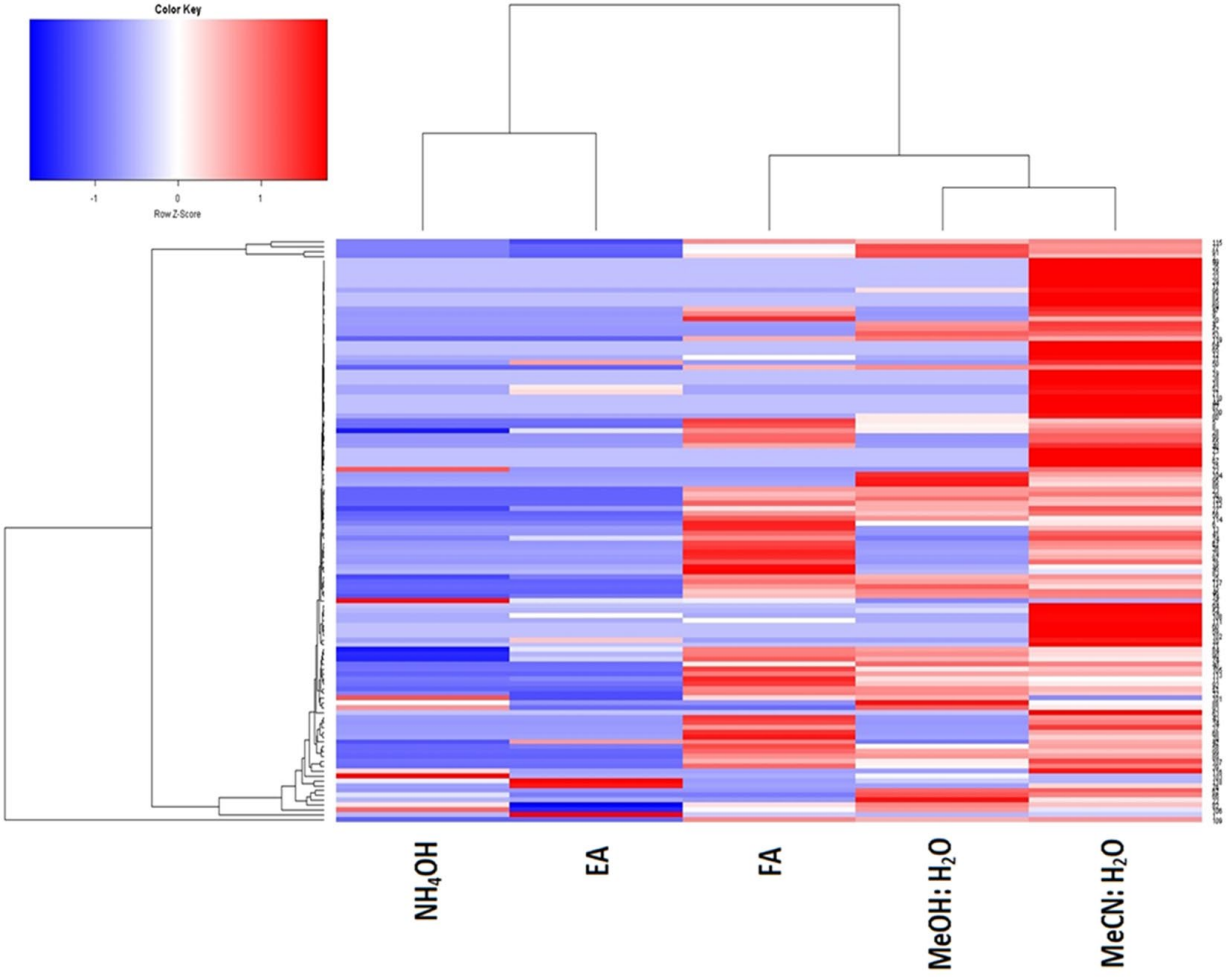
Unsupervised hierarchical clustering and heatmap of the identified metabolites in the extracted samples (rows) through different solvents (columns). NH_4_OH (**B**), ethyl acetate (**C**), Formic acid (**A**), methanol: water (**D**) acetonitrile: water (**E**). Cell color reflects metabolite relative content. The metabolites that correspond to the row numbers shown on the heat map listed in Supplementary Table 8.

Metabolite data obtained from the five extraction protocols were subjected to unsupervised hierarchical clustering to identify patterns and clustering of metabolite recovery generated from the different extraction methods. Figure 3 shows the clustering patterns of each metabolic extract based on the relative amount of each metabolite in the extracted solutions (blue show decrease and red show increase in the metabolite concentration).

The clustering information in Fig. 3 show the five extracts are differentiated based on the polarity of the different extraction solvents used in this study. Two clear clusters, as predicted, were therefore noticed corresponding to ammonium hydroxide (NH_4_OH) & ethyl acetate (cluster 1), and formic acid (FA), methanol: water, and acetonitrile: water (cluster 2). Taken together, the clustering showed that MeOH: water, MeCN:water and FA (0.2%) give closely related metabolite signature which is different from the closely related NH_4_OH and ethyl acetate solvent extraction group. This show that the solvent extraction method can identify novel metabolites that may not be detected through other methodologies. This information was also validated by subjecting the metabolite data to Principle Component Analysis (PCA) (Fig. S1) and Venn diagram (see Fig. 2). Also, PCA revealed that the metabolic information generated from the aqueous solutions were more similar (i.e. less variable) than the other solvents (Fig. S2).

### Pathways of MCF-7 breast cancer metabolome identified by GC-MS

The metabolites of (glycolysis, TCA and gluconeogenesis) were extracted and identified in most of the solvents used in this study, however, the concentration of the detected metabolites involved in these pathways were significantly affected by the polarity as well as the pH of the extraction solvents, for instance polar metabolites are more efficiently extracted in aqueous solution and the polarity of the metabolites is also affected by their ionization state which is pH and pKa dependent. However, more lipophilic metabolites of lipids and fatty acids tend to be more efficiently extracted in organic solvents (ethyl acetate), however, the ionization of these metabolites is significantly promoted in alkaline medium (NH_4_OH) which justify the presence of these metabolites in this aqueous solvent.

**Figure 4:**
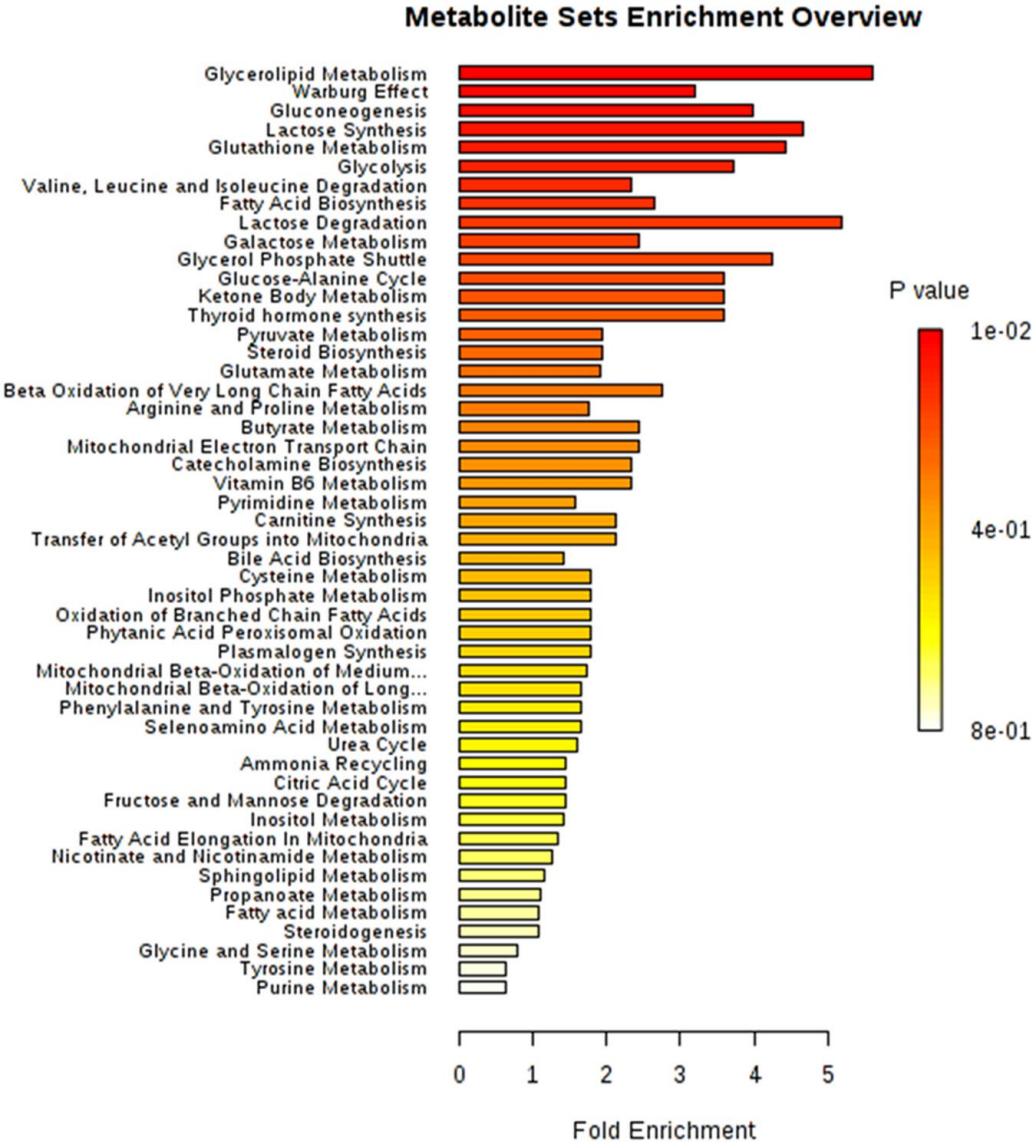
A diagram showing the MSEA for the identified metabolites in cluster 1: NH_4_OH and ethyl acetate.

MSEA results show enrichment in metabolic pathways for both extraction solvent groups with NH_4_OH and ethyl acetate showing higher enrichment of the Warburg effect (p = 0.03) as shown in Fig. 4 and Tables (S6 & S7). In fact, Warburg effect is the second most enriched pathway in metabolism.

**Figure 5:**
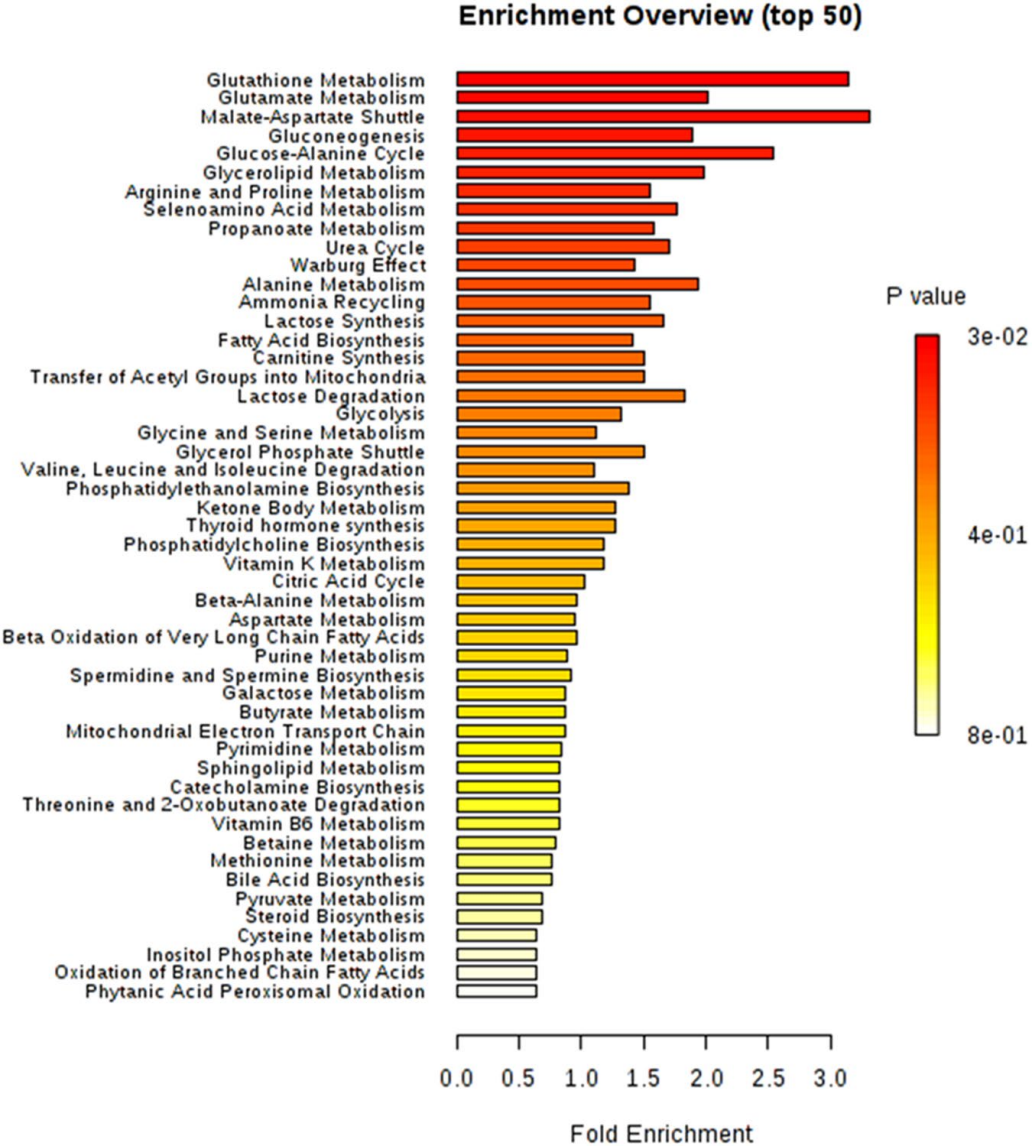
A diagram showing the MSEA of the identified metabolites in cluster 2: FA, MeOH: H_2_O and MeCN:H_2_O solvents.

However in the FA, MeOH: H_2_O and MeCN: H_2_O group the Warburg is in the top 10 (Fig. 5). For metabolic pathways the top enriched pathways are related to glycolysis, TCA and oxidative phosphorylation pathways, all of which are related to energy production and regulation and hence enhancement of the tumour growth.

### Pathways of MCF-7 breast cancer metabolome identified by GC-MS

Mapping of the metabolites from GC-MS to the genome SNP database, provided us with useful data regarding the possible genes involved with the metabolites. The enrichment pathways showed the involvement of Dynein cytoplasmic 2 heavy chain 1 gene (DYNC2H1), which is probably involved in protein transport via the chemi-osmotic potential and thus ATP production^38^. In addition other genes enriched included EVC: Ellis-van Creveld syndrome (EvC) and Weyers acrodental dysostosis which is linked to hedgehog pathway and is therefore related to Wnt signaling pathway involved in many cancers.

In addition, one of the genes identify through the rs1018503 SNP is NCOA6 (Nuclear Receptor Coactivator 6) which amongst its related pathways are BMAL1-CLOCK, NPAS2 that activates circadian gene expression^39^.

This in turn activate the transcription of AR (androgen receptor) which regulate KLK2 and KLK3 genes amongst others. Gene Ontology (GO) annotations related to NCOA6 gene include chromatin binding and transcription coactivator activity.

NCOA6 has bene shown to be associated with Breast Cancer cells^40^ which validates the finding from this study and show an interesting novel finding that relate metabolomics to specific genes related to breast cancer which may not be identified through genomic techniques and thus warrant further investigation.

## Discussion

Metabolomics is an important diagnostic tool in tackling the machinery of tumor progression. Human breast cancers have a broad array of causes that modulate growth and metastasis. Therefore, biomarkers identification involved in certain pathways might reveal intrinsic causes of cancer and chemotherapy resistance. In this study, the solvent extraction efficiency of metabolites in MCF-7 cancer cells was evaluated.

The extraction results showed that 0.2% formic acid and acetonitrile: water mixture solutions, have showed the best extraction efficiency, whereas solutions B, C, and D (Table 1) showed inferior to moderate extraction efficiency. These differences in metabolite extraction efficiency might be due to both polarity and pH values of the extraction solvents, as well as the pKa’s of the metabolites. Polarity of the extraction solutions decreases in following order: aqueous solutions > methanol > acetonitrile > ethyl acetate, whereas the pH value decreases according to this order: 0.2% ammonium hydroxide in water > ethyl acetate > methanol > acetonitrile >0.2% formic acid in water. Solution A (FA 0.2%) and E (acetonitrile) showed the highest extraction efficiency in terms of both metabolites detection and identification (111 and 120 metabolites were detected respectively). This can be explained by the biochemical properties of cancer cells themselves. Several studies have indicated that one of the characteristics that distinguishes cancer cells from normal cells is their low intracellular pH value^41,42^, indicating the existence of chemically stable cancer-cell-specific metabolites. Therefore, acidified extracellular media, i.e. solvent with lower pH value, which in our case is the extraction solution A, provided greater extraction yield, due to the ionization of basic metabolites at low pH values or the existence neutral and polar metabolites, thus enabling the optimal conditions for diffusion of cancer-cell-specific metabolites to occur in the aqueous acidic medium. Moreover, a similar rationale can explain the extraction efficiency of acetonitrile: water solution, which is a slightly basic and polar solution, favoring mainly the extraction of polar neutral and acidic metabolites in higher concentrations. Certain metabolites have been extracted when solution B (0.2% ammonium hydroxide) was employed and this can be explained by the dominant existence of ionized acidic metabolites at alkaline pH. However, the extracted metabolites did not provide any significant information about the specific metabolic pathways occurring in MCF-7 cells. Moreover, each of the chromatograms and fragmentation patterns of the extracted solutions needs to be profoundly investigated in terms of metabolite peak intensity and characterization to evaluate the solvent effect, reproducibility and extraction efficiency.

Inferiority of the extraction solution C is based on the fact that the metabolites are mainly polar in their nature and the extraction is favored in aqueous medium, which is not the case for ethyl acetate as an organic solvent. However, ribitol which is a major metabolite of the pentose phosphate pathway in MCF-7, was efficiently extracted in the solutions C and D.

Surprisingly, non-conventional metabolites (Stigmasterol and its metabolite) were identified in the MCF-7 cells, and these metabolites are not regularly found in healthy normal cells. In this specific case, the detection of Stigmasterol (phytoestrogen) and its’ metabolite implies an alteration in the cholesterol overall metabolism.

This study showed that mass spec can identify key metabolites that are associated with different diseases. In this case, it identified the NCOA6 gene as one of the genes linked to metabolites, which activates circadian gene expression and downstream-related pathways. However, this gene is not commonly associated with breast cancer but a recent paper^43^ showed that it regulates the expression of kallikreins 2 and 3 in human breast cancer cell lines and can therefore be thought of as low penetrance gene that can explain one of the environmental link to breast cancer.

In addition to that, all the metabolites identified showed link to glycolysis and therefore may shed light on how energy metabolism has been hijacked to encourage tumour progression and eventually metastasis in breast cancer. Other studies reported on similar phenomena in various cancers including breast^44^. However, this study showed that in addition to the energy metabolism pathways, it identified the Warburg effect as a key component in MCF-7 cell lines indicating that hypoxic conditions are key to its survival and proliferation. The Warburg effect can be induced by mitochondrial dysfunction or mutations in essential glycolytic enzymes such as 6-phosphofructo-1-kinase (PFK-1) triggered by hypoxia via the stimulation of the HIF-1 complex (hypoxia-inducible complex-1). HIF-1 complex is a transcriptional modulator that regulates the expression of most of hypoxia-related genes and is overexpressed in a variety of tumours. In addition, it is worth investigating the role of other genetic biomarkers identified through the GC-Mass spectroscopy data from this study, such as NCOA6 which may identify some of the environmental links to breast cancer such as stress or abnormal sleep patterns.

Taken together, using GC-Mass spectroscopy approach provides a different perspective in shedding light on the mechanism of breast cancer and identifying some of the genes that are associated with breast cancer pathogenesis (such as NCOA6) that could potentially be biomarkers for breast cancer.

## Conclusions

This study showed that GC-MS could identify key metabolites that are associated with metabolic disorders and proved that the extraction efficiency is not limited to one specific solvent, due to the differences in physicochemical properties of the extracted metabolites.

Among the five solvents that have been used to investigate the extraction efficiency in this study, the greatest extraction rate and diversity of metabolites was obtained by employing 0.2% formic acid (solvent A) and acetonitrile: water mixture (solvent E). The fact that solvent A and E provided high reproducibility, selectivity and sensitivity for the extracted metabolites, is encouraging to exploit this efficient extraction methodology in future quantitative investigations on MCF-7 cancer cells. Our future plan is therefore to investigate the effects of treating triple positive and triple negative MCF-7 cells with different anticancer agents to characterize malfunctioning metabolic pathways.

Furthermore, the majority of the identified metabolites are linked to glycolysis and therefore this may shed light on how energy metabolism could be hijacked to encourage tumour progression and eventually metastasis in breast cancer.

Moreover, in addition to the energy metabolism pathways, the MSEA identified Warburg effect as a key component in MCF-7 breast cancer cells indicating that hypoxic conditions play a role in their survival and proliferation. On the other hand, the ability of this extraction methodology to reveal Warburg effect, which has been previously reported in the literature, proves the efficiency, selectivity and applicability of the developed extraction methodology.

Finally, mapping of the identified metabolites to the genome SNP database, identified NCOA6 gene as a key component involved in the activation of circadian gene expression and downstream-related pathways.

## Materials and Methods

Metabolite extraction solvents were of LC-MS grade. Formic acid and methanol were obtained from Fisher Chemical (Loughborough, UK), whereas acetonitrile and ammonium hydroxide (30–33% grade) were obtained from Sigma Aldrich (Sr. Louis, MO, USA).

Reagents for derivatization included methoxyamine hydrochloride (98%) obtained from Sigma Aldrich, pyridine acquired from Merck (Darmstadt, Germany) and N-Methyl-N-(trimethylsilyl) trifluoroacetamide with 1% trimethylchlorosilane purchased from Sigma Aldrich.

As an internal standard for GC-MS analysis, Column Test Mix, containing methyl decanoate, methyl undecanoate, 2,3-butanediol, dicyclohexylamine, 2,6-dimethyleaniline, 2,6-dimethylphenol, nonanal, 1-octanol, undecane, decane, hexachlorobenzene dissolved in n-hexane manufactured by Varian (Walnut Creek, CA, USA) was used for mass calibration.

### Cell line and culture condition

The breast adenocarcinoma cells (MCF-7) were cultured in DMEM medium supplemented with 10% fetal calf serum and 1% penicillin/streptomycin solution and incubated at 37 °C in an atmosphere of 5% CO_2_. In preparation for an experiment, 3 × 10^6^ cells were cultured in each of 15 tissue culture flasks (175 cm^2^) and cells were incubated for three days. When the cells reach 80% confluency, they were collected by trypsinization, counted in a cell counter and each 3 × 10^6^ cells were suspended in 1 ml phosphate-buffered saline in an Eppendorf tube.

### Metabolite extraction and derivatization

A total of 5 triplicated MCF-7 cell culture samples were provided in Eppendorf vials dissolved in PBS, and stored at 4 °C for preservation purposes. Samples were centrifuged at 13000 rpm for 10 minutes at −4 °C. Supernatant was discarded, and cell pellets, each containing 3 million cells, were subjected to metabolomics analysis.

In order to evaluate the influence of the solvents on the extraction rate, samples were divided in five different extraction groups: A, 0.2% formic acid in water; B, 0.2% ammonium hydroxide in water; C, ethyl acetate; D, methanol/water (1:1, v/v); and E, acetonitrile/water (1:1, v/v).

Metabolic content extraction of the adenocarcinoma cells (MCF-7), applied in this research, was based on optimized method by our research group. Briefly, 300 µL of the extraction solvent was added to 3 million cell pellets, then vortexed for 2 minutes. To ensure the quantitative extraction of the metabolites, samples have been stored in ice for 1 hour, during which samples have been vortexed every 15 minutes. After this, cell insoluble matrix was centrifuged (13000 rpm, 10 minutes, −4 °C). Supernatant was collected and transferred to GC vial, then dried using EZ-2 Plus (GeneVac, Ipswich, UK) at 37 ± 1 °C. Amino acids, saccharides, fatty acids and steroids cannot be analysed directly by gas chromatography due to their high polarity and low volatility. Hence, it was necessary to derivatize them prior to the GC-MS analysis. Having said that, dry samples were dissolved in 25 µL of a solution consisting 20 mg/mL methoxyamine hydrochloride in pyridine, followed by vortexing for 2 minutes, and storing for at least 6 hours at 25 °C prior to the silylation step. Then 25 µL of MSTFA + 1% TMCS was added, followed by dissolving in 100 µL of pyridine, and vortexing for 2 minutes. For complete derivatization, it was necessary to incubate the samples at 50 °C for 30 minutes. Due to low volume, all of the samples have been immediately transferred to 200 µL microinserts, and analysed by GC-MS.

### Gas chromatography – mass spectrometric analysis of the samples

A GC/MS-QP 2010 Ultra System (Shimadzu, Kyoto, Japan) was employed for the metabolomic analysis, along with LabSolutions GC-MS software (v1.20). A Restek Rtx^®^ − 5ms column (30.0 m × 0.25 mm, 0.25 µm) was utilized for separation of the metabolites. Helium (99.9%) was utlized as the carrier gas at the flow rate of 1.0 mL/min. The initial oven temperature was set at 60 °C and was held at this temperature for 2 minutes, then raised to 310 °C by 50 °C/min and held at this temperature during the analysis. Both the auxiliary temperature at the interface and the ionization temperature were 250 °C. Metabolites were analysed in full scan mode within the range of 50–650 amu. Total volume of 10 µL was injected in splitless mode, by employing AOC-20i Auto Injector (Shimadzu, Kyoto, Japan). GC total ion chromatograms (TIC) and fragmentation patterns of the metabolites identified using NIST/EPA/NIH Mass Spectral Library (NIST 14). Run time for each sample was 43.67 min.

### Bioinformatics and functional genomic

Gas chromatography data was acquired as GCMS Data File (QGD) files and stored in text format. Mining of the metabolites data was carried out using various bioinformatics algorithms followed by mapping to genomics to acquire functional genomics data. All data including the raw QGD files, has been deposited to Metabolomics Workbench (https://www.metabolomicsworkbench.org). The data track id is 1759.

### Unsupervised hierarchical clustering

The mass signal from the GCMS data was manually filtered to remove unidentified ions from the table. Signals that belong to the same category of ions were grouped together and the data normalised based on the total peak height. The table with metabolic data from all extraction replicates generated from the five solvents (triplicate for extraction) was then assessed in detail. Principle Component Analysis (PCA) was implemented using R programming language (version 3.5.2) using the *prcomp* library. Unsupervised hierarchical clustering was carried out using R programming language to explore possible grouping and patterns in metabolic coverage generated from the five extraction solutions.

### MSEA – functional metabolomics

Metabolome coverage between the five different extracts was conducted using both MSEA and network‐based metabolic pathway analysis (MPA)^45^. MSEA tests, using over-representation analysis, the enrichment of metabolites in a metabolic pathway compared to the total of annotated metabolites in the same pathway. False Discovery Rate was calculated according to the Benjamini and Hochberg multiple testing correction statistical method^46^. Metaboanalyst^43^, was used to carry out the statistical analysis, MSEA and MPA. The enriched data was mapped to Single Nucleotide Polymorphism (SNP) databases to add biological meaning and aid in its interpretation.

## Supplementary information

Supplementary information
