# Supplementary material for: Comparative metabolomics of MCF-7 breast cancer cells using different extraction solvents assessed by mass spectroscopy
Source: Sci Rep. 2019 Sep 11;9:13126. doi: 10.1038/s41598-019-49509-y (PMC6739366; doi:10.1038/s41598-019-49509-y)
Supplement: Supplementary file 1 — Supplementary information [file 41598_2019_49509_MOESM1_ESM.docx]

**Comparative metabolomics of MCF-7 breast cancer cells using different extraction solvents assessed by mass spectroscopy**

Mohammad H. Semreen^1,2^*, Hasan Y. Alniss^1,2^, Stefan Grgic^2,3^, Raafat El-Awady^1,2^, Ahmed Almehdi^4^, Muath K. Mousa,^5^ Rifat Hamoudi^2,6,7^*

^1^College of Pharmacy, University of Sharjah, P.O. Box 27272, Sharjah, United Arab Emirates. ^2^Sharjah Institute for Medical Research, University of Sharjah, P.O. Box 27272, Sharjah, United Arab Emirates.

^3^Faculty of Medicine, Department of Pharmacy, University of Novi Sad, P.O Box 21001, Novi Sad, Serbia.

^4^department of Chemistry, College of Sciences, University of Sharjah, P.O. Box 27272, Sharjah, United Arab Emirates.

^5^ Research Institute of Science and Engineering, University of Sharjah, P.O. Box 27272, Sharjah, United Arab Emirates.

^6^College of Medicine, University of Sharjah, P.O. Box 27272, Sharjah, United Arab Emirates.

^7^Division of Surgery and Interventional Science, University College London, London, United Kingdom.

*Corresponding authors:

E-mail: address msemreen@sharjah.ac.ae

Department of Medicinal Chemistry, College of Pharmacy, University of Sharjah, P.O. Box. 27272, Sharjah, United Arab Emirates

and

E-mail address: rhamoudi@sharjah.ac.ae

College of Medicine, University of Sharjah, P.O. Box 27272, Sharjah, United Arab Emirates

**Supplementary Table 1**. List of extracted metabolites using extraction solvent A (0.2 % formic acid in water)

| **Identification** | **Retention time** | **Area under curve** | **PubChem ID** |
| --- | --- | --- | --- |
| Boric acid | 5.632 | 23321619 | 7628 |
| Lactic Acid | 7.189 | 135115766 | 107689 |
| Glycolic acid | 7.666 | 225444 | 757 |
| 1H-Azonine | 7.784 | 0 | 13287582 |
| L-Valine | 7.854 | 2448318 | 6287 |
| Alanine | 8.159 | 4899056 | 5950 |
| Ethylene glycol | 8.205 | 0 | 174 |
| Glycine | 8.477 | 2082755 | 750 |
| 3-Pyridinol | 8.777 | 730440 | 7971 |
| Silane | 8.992 | 0 | 23953 |
| L-Leucine | 9.202 | 11211540 | 6106 |
| 4-Hydroxybutanoic acid | 9.437 | 0 | 7302 |
| L-Proline | 9.565 | 4646866 | 145742 |
| L-Isoleucine | 9.622 | 4412840 | 6306 |
| Trisiloxane | 9.906 | 3735642 | 17860093 |
| N-Acetylaminoethanol | 10.449 | 0 | 14960462 |
| L-Valine | 10.574 | 0 | 6287 |
| Benzoic Acid | 11.05 | 0 | 243 |
| 3-Ethyl-3-methylheptadecane | 11.097 | 0 | 11536486 |
| Diethylene glycol | 11.14 | 879981 | 8117 |
| 4-Aminobutanoic acid | 11.402 | 0 | 119 |
| Glycerol | 11.626 | 0 | 753 |
| L-Threonine | 12.269 | 2599095 | 6288 |
| 3-Aminoisobutyric acid | 12.437 | 2686471 | 64956 |
| Butanedioic acid | 12.592 | 4746815 | 1110 |
| Cyclohexasiloxane | 12.9 | 2425750 | 9903713 |
| 2-Butenedioic acid | 13.056 | 1528801 | 444266 |
| Glyceric acid | 13.148 | 1205776 | 439194 |
| Myristic acid | 13.294 | 0 | 11005 |
| Ethylphosphonic acid | 13.379 | 2199159 | 204482 |
| 6-Dimethyl(trimethylsilyl)silyloxytetradecane | 14.396 | 157392 | 590061 |
| Thiodiglycol | 14.5 | 10610738 | 5447 |
| beta-Alanine | 14.768 | 0 | 239 |
| L-Aspartic acid | 14.814 | 13323823 | 5960 |
| 2,4'-Bipyridine | 15.158 | 0 | 68488 |
| L-Hydroxyproline | 15.707 | 8449003 | 5810 |
| Succinic acid | 15.826 | 8494069 | 1110 |
| 1-Hexanol | 16.004 | 0 | 8103 |
| Malic acid | 16.171 | 32721975 | 525 |
| Pyroglutamic acid | 16.336 | 5508543 | 7405 |
| (2-Ethoxyethoxy)acetic acid | 16.555 | 3588884 | 192850 |
| L-5-Oxoproline | 16.788 | 21049984 | 7405 |
| Acetoin | 17.079 | 0 | 179 |
| DL-Phenylalanine | 17.154 | 7999166 | 994 |
| 2,3,4-Trihydroxybutyric acid | 17.447 | 4707336 | 439535 |
| Pentanedioic acid | 17.854 | 734124 | 743 |
| L-Proline | 17.979 | 1226575 | 145742 |
| Amphetamine | 18.058 | 0 | 3007 |
| Pimelic acid | 18.147 | 0 | 385 |
| Triethanolamine | 18.625 | 13106735 | 7618 |
| Dodecanoic acid | 18.762 | 0 | 3893 |
| 2,2'-Sulfinyldiethanol | 19.174 | 0 | 18330 |
| Propanoic acid | 19.332 | 0 | 1032 |
| Levoglucosan | 20.279 | 2171042 | 2724705 |
| Erythro-Pentonic acid | 20.917 | 2934564 | 90658608 |
| Nonadecane | 21.08 | 0 | 12401 |
| Phosphoric acid | 21.284 | 24335810 | 1004 |
| Phosphorylethanolamine | 21.456 | 0 | 1015 |
| Citric acid | 22.214 | 8083869 | 311 |
| D-(+)-Ribono-1 | 22.395 | 0 | 111064 |
| Deoxyglucose | 22.481 | 0 | 108223 |
| Sulfurous acid | 22.686 | 0 | 1100 |
| beta-D-Galactofuranoside | 22.755 | 0 | 22215147 |
| D-Pinitol | 22.944 | 0 | 164619 |
| D-(-)-Tagatose | 23.291 | 0 | 92092 |
| Glucopyranose | 23.389 | 21798540 | 5793 |
| Pentadecanoic acid | 23.586 | 0 | 13849 |
| d-Glucose, 2,3,4,5,6-pentakis-O-(trimethylsilyl) | 23.656 | 22356914 | 522260 |
| L-Tyrosine | 23.843 | 0 | 6057 |
| d-Mannose, 2,3,4,5,6-pentakis-O-(trimethylsilyl) | 23.947 | 0 | 5378916 |
| Diisooctyl phthalate | 24.207 | 0 | 33934 |
| Octasiloxane | 24.346 | 7455557 | 21881078 |
| Glucopyranose | 24.856 | 0 | 5793 |
| Palmitic Acid | 25.072 | 172160405 | 985 |
| D-Gluconic acid | 25.199 | 5370617 | 10690 |
| 2(1H)-Pyrimidinone | 25.405 | 8018536 | 68401 |
| Scyllo-Inositol | 25.617 | 0 | 892 |
| Octadecane | 25.678 | 0 | 11635 |
| Heptadecanoic acid | 26.451 | 8018536 | 10465 |
| Octadecane-1,2-diol | 26.543 | 9552163 | 89314 |
| Cyclopentanemethanol | 26.833 | 0 | 77195 |
| 2-Methyltetracosane | 27.071 | 0 | 527459 |
| Spermine | 27.32 | 27.304 | 1103 |
| Heptadecyl acetate | 27.386 | 0 | 69967 |
| 9,12-Octadecadienoic acid (Z,Z) | 27.462 | 21675714 | 5379173 |
| Oleic Acid | 27.577 | 9953575 | 445639 |
| Stearic acid | 27.825 | 130881548 | 5281 |
| Phosphoric acid, bis(trimethylsilyl) 2,3-bis[(trimethylsilyl)oxy]propyl ester | 27.924 | 8811015 | 553774 |
| N,N-Dimethyldodecanamide | 28.162 | 0 | 18159 |
| 1,1,1,5,7,7,7-Heptamethyl-3,3-bis(trimethylsiloxy)tetrasiloxane | 28.476 | 0 | 6329081 |
| 9,12-Octadecadienoic acid (Z,Z) | 28.65 | 0 | 5379173 |
| 18-Methyl-nonadecanol, trimethylsilyl ether | 29.156 | 0 | 91743660 |
| D-Galactofuranose, 1,2,3,5-tetrakis-O-(trimethylsilyl)-, bis(trimethylsilyl) phosphate | 29.552 | 7305680 | 553776 |
| d-Glucose, 2,3,4,5-tetrakis-O-(trimethylsilyl)-, o-methyloxime, 6-[bis(trimethylsilyl) phosphate | 29.847 | 2295652 | 90473467 |
| 1-Monomyristin | 29.97 | 20073580 | 79050 |
| Sebacic acid | 30.161 | 0 | 5192 |
| Octasiloxane, 1,1,3,3,5,5,7,7,9,9,11,11,13,13,15,15-hexadecamethyl | 30.286 | 10707270 | 6329087 |
| Eicosanoic acid | 30.326 | 0 | 10467 |
| Phenol, 2,2'-methylenebis[6-(1,1-dimethylethyl)-4-methyl | 30.641 | 0 | 8398 |
| N,N-Dimethyldodecanamide | 30.802 | 0 | 18159 |
| Myo-Inositol, 1,3,4,5,6-pentakis-O-(trimethylsilyl)-, bis(trimethylsilyl) phosphate | 30.869 | 11647390 | 553143 |
| 2-Methyl-6-tert-butylphenol | 31.107 | 61793387 | 16678 |
| benzene, 1,1'-methylenebis[3-(1,1-dimethylethyl)-5-methyl-4-[(trimethylsilyl)oxy] | 31.875 | 28567659 | 91697106 |
| Heptamethyl-3,3-bis(trimethylsiloxy)tetrasiloxane | 31.982 | 0 | 6329081 |
| 1-Monopalmitin | 32.358 | 748222288 | 3084463 |
| 5-Methyluridine | 33.045 | 0 | 445408 |
| Adenosine | 33.269 | 2851465 | 60961 |
| Heptadecanoic acid, glycerine-(1)-monoester, bis-O-trimethylsilyl | 33.364 | 2675410 | 633500 |
| Heptamethyl-3,3-bis(trimethylsiloxy)tetrasiloxane | 33.555 | 8216347 | 6329081 |
| 2-Monostearin | 34.053 | 3587058 | 79075 |
| Glycerol monostearate | 34.472 | 352315760 | 79075 |
| 9-Octadecenamide | 34.716 | 0 | 5283387 |
| 2,3-Dihydroxypropyl icosanoate | 36.416 | 4720541 | 3246944 |
| Cholesterol | 38.429 | 3066638 | 11025495 |
| Stigmasterol | 39.76 | 658466 | 5280794 |
| Stigmast-5-ene | 40.3 | 2194388 | 21771614 |

**Supplementary Table 2.** List of extracted metabolites using extraction solvent B (0.2 % ammonium hydroxide in water)

| **Identification** | **Retention time** | **Area under curve** | **PubChem ID** |
| --- | --- | --- | --- |
| Boric acid | 5.701 | 0 | 7628 |
| Lactic Acid | 7.189 | 21342253 | 107689 |
| Glycolic acid | 7.666 | 0 | 757 |
| 1H-Azonine | 7.784 | 0 | 13287582 |
| L-Valine | 7.854 | 0 | 6287 |
| Alanine | 8.159 | 0 | 5950 |
| Ethylene glycol | 8.205 | 0 | 174 |
| Glycine | 8.477 | 0 | 750 |
| 3-Pyridinol | 8.777 | 0 | 7971 |
| Silane | 8.992 | 0 | 23953 |
| L-Leucine | 9.202 | 0 | 6106 |
| 4-Hydroxybutanoic acid | 9.437 | 0 | 7302 |
| L-Proline | 9.565 | 0 | 145742 |
| L-Isoleucine | 9.622 | 0 | 6306 |
| Trisiloxane | 9.906 | 0 | 17860093 |
| N-Acetylaminoethanol | 10.449 | 0 | 14960462 |
| L-Valine | 10.574 | 0 | 6287 |
| Benzoic Acid | 11.05 | 0 | 243 |
| 3-Ethyl-3-methylheptadecane | 11.097 | 0 | 11536486 |
| Diethylene glycol | 11.14 | 0 | 8117 |
| 4-Aminobutanoic acid | 11.402 | 0 | 119 |
| Glycerol | 11.626 | 9011155 | 753 |
| L-Threonine | 12.269 | 0 | 6288 |
| 3-Aminoisobutyric acid | 12.437 | 0 | 64956 |
| Butanedioic acid | 12.592 | 0 | 1110 |
| Cyclohexasiloxane | 12.9 | 8928220 | 9903713 |
| 2-Butenedioic acid | 13.056 | 0 | 444266 |
| Glyceric acid | 13.148 | 0 | 439194 |
| Myristic acid | 13.294 | 0 | 11005 |
| Ethylphosphonic acid | 13.379 | 0 | 204482 |
| 6-Dimethyl(trimethylsilyl)silyloxytetradecane | 14.396 | 0 | 590061 |
| Thiodiglycol | 14.5 | 1784213 | 5447 |
| beta-Alanine | 14.768 | 0 | 239 |
| L-Aspartic acid | 14.814 | 0 | 5960 |
| 2,4'-Bipyridine | 15.158 | 0 | 68488 |
| L-Hydroxyproline | 15.707 | 0 | 5810 |
| Succinic acid | 15.826 | 0 | 1110 |
| 1-Hexanol | 16.004 | 0 | 8103 |
| Malic acid | 16.171 | 0 | 525 |
| Pyroglutamic acid | 16.336 | 0 | 7405 |
| (2-Ethoxyethoxy)acetic acid | 16.555 | 0 | 192850 |
| L-5-Oxoproline | 16.788 | 0 | 7405 |
| Acetoin | 17.079 | 0 | 179 |
| DL-Phenylalanine | 17.154 | 0 | 994 |
| 2,3,4-Trihydroxybutyric acid | 17.447 | 0 | 439535 |
| Pentanedioic acid | 17.854 | 0 | 743 |
| L-Proline | 17.979 | 0 | 145742 |
| Amphetamine | 18.058 | 0 | 3007 |
| Pimelic acid | 18.147 | 0 | 385 |
| Triethanolamine | 18.625 | 0 | 7618 |
| Dodecanoic acid | 18.762 | 0 | 3893 |
| 2,2'-Sulfinyldiethanol | 19.174 | 0 | 18330 |
| Propanoic acid | 19.332 | 0 | 1032 |
| Levoglucosan | 20.279 | 0 | 2724705 |
| Erythro-Pentonic acid | 20.917 | 0 | 90658608 |
| Nonadecane | 21.08 | 0 | 12401 |
| Phosphoric acid | 21.284 | 0 | 1004 |
| Phosphorylethanolamine | 21.456 | 0 | 1015 |
| Citric acid | 22.214 | 0 | 311 |
| D-(+)-Ribono-1 | 22.395 | 0 | 111064 |
| Deoxyglucose | 22.481 | 0 | 108223 |
| Sulfurous acid | 22.686 | 0 | 1100 |
| beta-D-Galactofuranoside | 22.755 | 0 | 22215147 |
| D-Pinitol | 22.944 | 14634505 | 164619 |
| D-(-)-Tagatose | 23.291 | 0 | 92092 |
| Glucopyranose | 23.389 | 0 | 5793 |
| Pentadecanoic acid | 23.586 | 0 | 13849 |
| d-Glucose, 2,3,4,5,6-pentakis-O-(trimethylsilyl) | 23.656 | 0 | 522260 |
| L-Tyrosine | 23.843 | 0 | 6057 |
| d-Mannose, 2,3,4,5,6-pentakis-O-(trimethylsilyl) | 23.947 | 3278546 | 5378916 |
| Diisooctyl phthalate | 24.207 | 0 | 33934 |
| Octasiloxane | 24.346 | 0 | 21881078 |
| Glucopyranose | 24.856 | 0 | 5793 |
| Palmitic Acid | 25.072 | 48574221 | 985 |
| D-Gluconic acid | 25.199 | 0 | 10690 |
| 2(1H)-Pyrimidinone | 25.405 | 0 | 68401 |
| Scyllo-Inositol | 25.617 | 0 | 892 |
| Octadecane | 25.678 | 0 | 11635 |
| Heptadecanoic acid | 26.451 | 0 | 10465 |
| Octadecane-1,2-diol | 26.543 | 0 | 89314 |
| Cyclopentanemethanol | 26.833 | 0 | 77195 |
| 2-Methyltetracosane | 27.071 | 0 | 527459 |
| Spermine | 27.32 | 0 | 1103 |
| Heptadecyl acetate | 27.386 | 4307192 | 69967 |
| 9,12-Octadecadienoic acid (Z,Z) | 27.462 | 0 | 5379173 |
| Oleic Acid | 27.577 | 0 | 445639 |
| Stearic acid | 27.825 | 36067511 | 5281 |
| Phosphoric acid, bis(trimethylsilyl) 2,3-bis[(trimethylsilyl)oxy]propyl ester | 27.924 | 0 | 553774 |
| N,N-Dimethyldodecanamide | 28.162 | 7140680 | 18159 |
| 1,1,1,5,7,7,7-Heptamethyl-3,3-bis(trimethylsiloxy)tetrasiloxane | 28.476 | 0 | 6329081 |
| 9,12-Octadecadienoic acid (Z,Z) | 28.65 | 0 | 5379173 |
| 18-Methyl-nonadecanol, trimethylsilyl ether | 29.156 | 0 | 91743660 |
| D-Galactofuranose, 1,2,3,5-tetrakis-O-(trimethylsilyl)-, bis(trimethylsilyl) phosphate | 29.552 | 0 | 553776 |
| d-Glucose, 2,3,4,5-tetrakis-O-(trimethylsilyl)-, o-methyloxime, 6-[bis(trimethylsilyl) phosphate | 29.847 | 0 | 90473467 |
| 1-Monomyristin | 29.97 | 0 | 79050 |
| Sebacic acid | 30.161 | 0 | 5192 |
| Octasiloxane, 1,1,3,3,5,5,7,7,9,9,11,11,13,13,15,15-hexadecamethyl | 30.286 | 15057648 | 6329087 |
| Eicosanoic acid | 30.326 | 0 | 10467 |
| Phenol, 2,2'-methylenebis[6-(1,1-dimethylethyl)-4-methyl | 30.641 | 53356073 | 8398 |
| N,N-Dimethyldodecanamide | 30.802 | 0 | 18159 |
| Myo-Inositol, 1,3,4,5,6-pentakis-O-(trimethylsilyl)-, bis(trimethylsilyl) phosphate | 30.869 | 0 | 553143 |
| 2-Methyl-6-tert-butylphenol | 31.107 | 108732894 | 16678 |
| benzene, 1,1'-methylenebis[3-(1,1-dimethylethyl)-5-methyl-4-[(trimethylsilyl)oxy] | 31.875 | 0 | 91697106 |
| Heptamethyl-3,3-bis(trimethylsiloxy)tetrasiloxane | 31.982 | 0 | 6329081 |
| 1-Monopalmitin | 32.358 | 0 | 3084463 |
| 5-Methyluridine | 33.045 | 0 | 445408 |
| Adenosine | 33.269 | 0 | 60961 |
| Heptadecanoic acid, glycerine-(1)-monoester, bis-O-trimethylsilyl | 33.364 | 0 | 633500 |
| Heptamethyl-3,3-bis(trimethylsiloxy)tetrasiloxane | 33.555 | 0 | 6329081 |
| 2-Monostearin | 34.053 | 0 | 79075 |
| Glycerol monostearate | 34.472 | 71396128 | 79075 |
| 9-Octadecenamide | 34.716 | 22103423 | 5283387 |
| 2,3-Dihydroxypropyl icosanoate | 36.416 | 0 | 3246944 |
| Cholesterol | 38.429 | 17827559 | 11025495 |
| Stigmasterol | 39.76 | 0 | 5280794 |
| Stigmast-5-ene | 40.3 | 0 | 21771614 |

**Supplementary Table 3**. List of extracted metabolites using extraction solvent C (ethyl acetate)

| **Identification** | **Retention time** | **Area under curve** | **PubChem ID** |
| --- | --- | --- | --- |
| Boric acid | 5.701 | 384617821 | 7628 |
| Lactic Acid | 7.189 | 0 | 107689 |
| Glycolic acid | 7.666 | 0 | 757 |
| 1H-Azonine | 7.784 | 0 | 13287582 |
| L-Valine | 7.854 | 0 | 6287 |
| Alanine | 8.159 | 0 | 5950 |
| Ethylene glycol | 8.205 | 0 | 174 |
| Glycine | 8.477 | 0 | 750 |
| 3-Pyridinol | 8.777 | 0 | 7971 |
| Silane | 8.992 | 7024276 | 23953 |
| L-Leucine | 9.202 | 400788 | 6106 |
| 4-Hydroxybutanoic acid | 9.437 | 0 | 7302 |
| L-Proline | 9.565 | 0 | 145742 |
| L-Isoleucine | 9.622 | 664503 | 6306 |
| Trisiloxane | 9.906 | 0 | 17860093 |
| N-Acetylaminoethanol | 10.449 | 0 | 14960462 |
| L-Valine | 10.574 | 0 | 6287 |
| Benzoic Acid | 11.05 | 0 | 243 |
| 3-Ethyl-3-methylheptadecane | 11.097 | 0 | 11536486 |
| Diethylene glycol | 11.14 | 0 | 8117 |
| 4-Aminobutanoic acid | 11.402 | 0 | 119 |
| Glycerol | 11.626 | 4358175 | 753 |
| L-Threonine | 12.269 | 0 | 6288 |
| 3-Aminoisobutyric acid | 12.437 | 992691 | 64956 |
| Butanedioic acid | 12.592 | 845520 | 1110 |
| Cyclohexasiloxane | 12.9 | 2261580 | 9903713 |
| 2-Butenedioic acid | 13.056 | 582703 | 444266 |
| Glyceric acid | 13.148 | 677598 | 439194 |
| Myristic acid | 13.294 | 0 | 11005 |
| Ethylphosphonic acid | 13.379 | 0 | 204482 |
| 6-Dimethyl(trimethylsilyl)silyloxytetradecane | 14.396 | 0 | 590061 |
| Thiodiglycol | 14.5 | 0 | 5447 |
| beta-Alanine | 14.768 | 0 | 239 |
| L-Aspartic acid | 14.814 | 0 | 5960 |
| 2,4'-Bipyridine | 15.158 | 0 | 68488 |
| L-Hydroxyproline | 15.707 | 0 | 5810 |
| Succinic acid | 15.826 | 0 | 1110 |
| 1-Hexanol | 16.004 | 0 | 8103 |
| Malic acid | 16.171 | 0 | 525 |
| Pyroglutamic acid | 16.336 | 0 | 7405 |
| (2-Ethoxyethoxy)acetic acid | 16.555 | 0 | 192850 |
| L-5-Oxoproline | 16.788 | 20064518 | 7405 |
| Acetoin | 17.079 | 0 | 179 |
| DL-Phenylalanine | 17.154 | 0 | 994 |
| 2,3,4-Trihydroxybutyric acid | 17.447 | 0 | 439535 |
| Pentanedioic acid | 17.854 | 0 | 743 |
| L-Proline | 17.979 | 0 | 145742 |
| Amphetamine | 18.058 | 0 | 3007 |
| Pimelic acid | 18.147 | 348700 | 385 |
| Triethanolamine | 18.625 | 0 | 7618 |
| Dodecanoic acid | 18.762 | 821367 | 3893 |
| 2,2'-Sulfinyldiethanol | 19.174 | 0 | 18330 |
| Propanoic acid | 19.332 | 0 | 1032 |
| Levoglucosan | 20.279 | 0 | 2724705 |
| Erythro-Pentonic acid | 20.917 | 0 | 90658608 |
| Nonadecane | 21.08 | 0 | 12401 |
| Phosphoric acid | 21.284 | 1494950 | 1004 |
| Phosphorylethanolamine | 21.456 | 0 | 1015 |
| Citric acid | 22.214 | 0 | 311 |
| D-(+)-Ribono-1 | 22.395 | 0 | 111064 |
| Deoxyglucose | 22.481 | 0 | 108223 |
| Sulfurous acid | 22.686 | 0 | 1100 |
| beta-D-Galactofuranoside | 22.755 | 0 | 22215147 |
| D-Pinitol | 22.944 | 0 | 164619 |
| D-(-)-Tagatose | 23.291 | 0 | 92092 |
| Glucopyranose | 23.389 | 23.356 | 5793 |
| Pentadecanoic acid | 23.586 | 0 | 13849 |
| d-Glucose, 2,3,4,5,6-pentakis-O-(trimethylsilyl) | 23.656 | 0 | 522260 |
| L-Tyrosine | 23.843 | 913608 | 6057 |
| d-Mannose, 2,3,4,5,6-pentakis-O-(trimethylsilyl) | 23.947 | 0 | 5378916 |
| Diisooctyl phthalate | 24.207 | 0 | 33934 |
| Octasiloxane | 24.346 | 3339048 | 21881078 |
| Glucopyranose | 24.856 | 39671827 | 5793 |
| Palmitic Acid | 25.072 | 21900783 | 985 |
| D-Gluconic acid | 25.199 | 0 | 10690 |
| 2(1H)-Pyrimidinone | 25.405 | 0 | 68401 |
| Scyllo-Inositol | 25.617 | 0 | 892 |
| Octadecane | 25.678 | 0 | 11635 |
| Heptadecanoic acid | 26.451 | 0 | 10465 |
| Octadecane-1,2-diol | 26.543 | 5427308 | 89314 |
| Cyclopentanemethanol | 26.833 | 0 | 77195 |
| 2-Methyltetracosane | 27.071 | 0 | 527459 |
| Spermine | 27.32 | 0 | 1103 |
| Heptadecyl acetate | 27.386 | 0 | 69967 |
| 9,12-Octadecadienoic acid (Z,Z) | 27.462 | 0 | 5379173 |
| Oleic Acid | 27.577 | 4107097 | 445639 |
| Stearic acid | 27.825 | 16141552 | 5281 |
| Phosphoric acid, bis(trimethylsilyl) 2,3-bis[(trimethylsilyl)oxy]propyl ester | 27.924 | 0 | 553774 |
| N,N-Dimethyldodecanamide | 28.162 | 0 | 18159 |
| 1,1,1,5,7,7,7-Heptamethyl-3,3-bis(trimethylsiloxy)tetrasiloxane | 28.476 | 0 | 6329081 |
| 9,12-Octadecadienoic acid (Z,Z) | 28.65 | 0 | 5379173 |
| 18-Methyl-nonadecanol, trimethylsilyl ether | 29.156 | 0 | 91743660 |
| D-Galactofuranose, 1,2,3,5-tetrakis-O-(trimethylsilyl)-, bis(trimethylsilyl) phosphate | 29.552 | 0 | 553776 |
| d-Glucose, 2,3,4,5-tetrakis-O-(trimethylsilyl)-, o-methyloxime, 6-[bis(trimethylsilyl) phosphate | 29.847 | 0 | 90473467 |
| 1-Monomyristin | 29.97 | 0 | 79050 |
| Sebacic acid | 30.161 | 0 | 5192 |
| Octasiloxane, 1,1,3,3,5,5,7,7,9,9,11,11,13,13,15,15-hexadecamethyl | 30.286 | 3216188 | 6329087 |
| Eicosanoic acid | 30.326 | 0 | 10467 |
| Phenol, 2,2'-methylenebis[6-(1,1-dimethylethyl)-4-methyl | 30.641 | 0 | 8398 |
| N,N-Dimethyldodecanamide | 30.802 | 0 | 18159 |
| Myo-Inositol, 1,3,4,5,6-pentakis-O-(trimethylsilyl)-, bis(trimethylsilyl) phosphate | 30.869 | 0 | 553143 |
| 2-Methyl-6-tert-butylphenol | 31.107 | 0 | 16678 |
| benzene, 1,1'-methylenebis[3-(1,1-dimethylethyl)-5-methyl-4-[(trimethylsilyl)oxy] | 31.875 | 0 | 91697106 |
| Heptamethyl-3,3-bis(trimethylsiloxy)tetrasiloxane | 31.982 | 2683405 | 6329081 |
| 1-Monopalmitin | 32.358 | 430815 | 3084463 |
| 5-Methyluridine | 33.045 | 0 | 445408 |
| Adenosine | 33.269 | 0 | 60961 |
| Heptadecanoic acid, glycerine-(1)-monoester, bis-O-trimethylsilyl | 33.364 | 0 | 633500 |
| Heptamethyl-3,3-bis(trimethylsiloxy)tetrasiloxane | 33.555 | 0 | 6329081 |
| 2-Monostearin | 34.053 | 0 | 79075 |
| Glycerol monostearate | 34.472 | 0 | 79075 |
| 9-Octadecenamide | 34.716 | 0 | 5283387 |
| 2,3-Dihydroxypropyl icosanoate | 36.416 | 0 | 3246944 |
| Cholesterol | 38.429 | 70835367 | 11025495 |
| Stigmasterol | 39.76 | 0 | 5280794 |
| Stigmast-5-ene | 40.3 | 0 | 21771614 |

**Supplementary Table 4.** List of extracted metabolites using extraction solvent D (methanol/water)

| **Identification** | **Retention time** | **Area under curve** | **PubChem ID** |
| --- | --- | --- | --- |
| Boric acid | 5.701 | 0 | 7628 |
| Lactic Acid | 7.189 | 228585341 | 107689 |
| Glycolic acid | 7.666 | 266851 | 757 |
| 1H-Azonine | 7.784 | 710926 | 13287582 |
| L-Valine | 7.854 | 1184688 | 6287 |
| Alanine | 8.159 | 1696802 | 5950 |
| Ethylene glycol | 8.205 | 0 | 174 |
| Glycine | 8.477 | 1032492 | 750 |
| 3-Pyridinol | 8.777 | 0 | 7971 |
| Silane | 8.992 | 0 | 23953 |
| L-Leucine | 9.202 | 5302656 | 6106 |
| 4-Hydroxybutanoic acid | 9.437 | 0 | 7302 |
| L-Proline | 9.565 | 0 | 145742 |
| L-Isoleucine | 9.622 | 5206709 | 6306 |
| Trisiloxane | 9.906 | 4673349 | 17860093 |
| N-Acetylaminoethanol | 10.449 | 0 | 14960462 |
| L-Valine | 10.574 | 0 | 6287 |
| Benzoic Acid | 11.05 | 0 | 243 |
| 3-Ethyl-3-methylheptadecane | 11.097 | 0 | 11536486 |
| Diethylene glycol | 11.14 | 0 | 8117 |
| 4-Aminobutanoic acid | 11.402 | 0 | 119 |
| Glycerol | 11.626 | 97130865 | 753 |
| L-Threonine | 12.269 | 2599095 | 6288 |
| 3-Aminoisobutyric acid | 12.437 | 0 | 64956 |
| Butanedioic acid | 12.592 | 4151836 | 1110 |
| Cyclohexasiloxane | 12.9 | 0 | 9903713 |
| 2-Butenedioic acid | 13.056 | 1871099 | 444266 |
| Glyceric acid | 13.148 | 862986 | 439194 |
| Myristic acid | 13.294 | 0 | 11005 |
| Ethylphosphonic acid | 13.379 | 2564961 | 204482 |
| 6-Dimethyl(trimethylsilyl)silyloxytetradecane | 14.396 | 0 | 590061 |
| Thiodiglycol | 14.5 | 10115886 | 5447 |
| beta-Alanine | 14.768 | 0 | 239 |
| L-Aspartic acid | 14.814 | 0 | 5960 |
| 2,4'-Bipyridine | 15.158 | 0 | 68488 |
| L-Hydroxyproline | 15.707 | 0 | 5810 |
| Succinic acid | 15.826 | 0 | 1110 |
| 1-Hexanol | 16.004 | 0 | 8103 |
| Malic acid | 16.171 | 17389740 | 525 |
| Pyroglutamic acid | 16.336 | 9582395 | 7405 |
| (2-Ethoxyethoxy)acetic acid | 16.555 | 0 | 192850 |
| L-5-Oxoproline | 16.788 | 2630123 | 7405 |
| Acetoin | 17.079 | 0 | 179 |
| DL-Phenylalanine | 17.154 | 0 | 994 |
| 2,3,4-Trihydroxybutyric acid | 17.447 | 5996779 | 439535 |
| Pentanedioic acid | 17.854 | 0 | 743 |
| L-Proline | 17.979 | 0 | 145742 |
| Amphetamine | 18.058 | 0 | 3007 |
| Pimelic acid | 18.147 | 0 | 385 |
| Triethanolamine | 18.625 | 12976871 | 7618 |
| Dodecanoic acid | 18.762 | 0 | 3893 |
| 2,2'-Sulfinyldiethanol | 19.174 | 985497 | 18330 |
| Propanoic acid | 19.332 | 985497 | 1032 |
| Levoglucosan | 20.279 | 0 | 2724705 |
| Erythro-Pentonic acid | 20.917 | 2455636 | 90658608 |
| Nonadecane | 21.08 | 727665 | 12401 |
| Phosphoric acid | 21.284 | 11923779 | 1004 |
| Phosphorylethanolamine | 21.456 | 0 | 1015 |
| Citric acid | 22.214 | 0 | 311 |
| D-(+)-Ribono-1 | 22.395 | 60622872 | 111064 |
| Deoxyglucose | 22.481 | 0 | 108223 |
| Sulfurous acid | 22.686 | 0 | 1100 |
| beta-D-Galactofuranoside | 22.755 | 0 | 22215147 |
| D-Pinitol | 22.944 | 45401248 | 164619 |
| D-(-)-Tagatose | 23.291 | 0 | 92092 |
| Glucopyranose | 23.389 | 23.386 | 5793 |
| Pentadecanoic acid | 23.586 | 0 | 13849 |
| d-Glucose, 2,3,4,5,6-pentakis-O-(trimethylsilyl) | 23.656 | 0 | 522260 |
| L-Tyrosine | 23.843 | 0 | 6057 |
| d-Mannose, 2,3,4,5,6-pentakis-O-(trimethylsilyl) | 23.947 | 0 | 5378916 |
| Diisooctyl phthalate | 24.207 | 0 | 33934 |
| Octasiloxane | 24.346 | 5875992 | 21881078 |
| Glucopyranose | 24.856 | 0 | 5793 |
| Palmitic Acid | 25.072 | 390605918 | 985 |
| D-Gluconic acid | 25.199 | 0 | 10690 |
| 2(1H)-Pyrimidinone | 25.405 | 0 | 68401 |
| Scyllo-Inositol | 25.617 | 616640 | 892 |
| Octadecane | 25.678 | 0 | 11635 |
| Heptadecanoic acid | 26.451 | 0 | 10465 |
| Octadecane-1,2-diol | 26.543 | 8676122 | 89314 |
| Cyclopentanemethanol | 26.833 | 0 | 77195 |
| 2-Methyltetracosane | 27.071 | 2711590 | 527459 |
| Spermine | 27.32 | 0 | 1103 |
| Heptadecyl acetate | 27.386 | 14309998 | 69967 |
| 9,12-Octadecadienoic acid (Z,Z) | 27.462 | 20762372 | 5379173 |
| Oleic Acid | 27.577 | 9608737 | 445639 |
| Stearic acid | 27.825 | 263133932 | 5281 |
| Phosphoric acid, bis(trimethylsilyl) 2,3-bis[(trimethylsilyl)oxy]propyl ester | 27.924 | 5193028 | 553774 |
| N,N-Dimethyldodecanamide | 28.162 | 8369157 | 18159 |
| 1,1,1,5,7,7,7-Heptamethyl-3,3-bis(trimethylsiloxy)tetrasiloxane | 28.476 | 0 | 6329081 |
| 9,12-Octadecadienoic acid (Z,Z) | 28.65 | 4011389 | 5379173 |
| 18-Methyl-nonadecanol, trimethylsilyl ether | 29.156 | 385256 | 91743660 |
| D-Galactofuranose, 1,2,3,5-tetrakis-O-(trimethylsilyl)-, bis(trimethylsilyl) phosphate | 29.552 | 0 | 553776 |
| d-Glucose, 2,3,4,5-tetrakis-O-(trimethylsilyl)-, o-methyloxime, 6-[bis(trimethylsilyl) phosphate | 29.847 | 0 | 90473467 |
| 1-Monomyristin | 29.97 | 16199139 | 79050 |
| Sebacic acid | 30.161 | 0 | 5192 |
| Octasiloxane, 1,1,3,3,5,5,7,7,9,9,11,11,13,13,15,15-hexadecamethyl | 30.286 | 12003544 | 6329087 |
| Eicosanoic acid | 30.326 | 0 | 10467 |
| Phenol, 2,2'-methylenebis[6-(1,1-dimethylethyl)-4-methyl | 30.641 | 11653162 | 8398 |
| N,N-Dimethyldodecanamide | 30.802 | 4703240 | 18159 |
| Myo-Inositol, 1,3,4,5,6-pentakis-O-(trimethylsilyl)-, bis(trimethylsilyl) phosphate | 30.869 | 6007297 | 553143 |
| 2-Methyl-6-tert-butylphenol | 31.107 | 95799753 | 16678 |
| benzene, 1,1'-methylenebis[3-(1,1-dimethylethyl)-5-methyl-4-[(trimethylsilyl)oxy] | 31.875 | 20333567 | 91697106 |
| Heptamethyl-3,3-bis(trimethylsiloxy)tetrasiloxane | 31.982 | 0 | 6329081 |
| 1-Monopalmitin | 32.358 | 655278547 | 3084463 |
| 5-Methyluridine | 33.045 | 0 | 445408 |
| Adenosine | 33.269 | 0 | 60961 |
| Heptadecanoic acid, glycerine-(1)-monoester, bis-O-trimethylsilyl | 33.364 | 2047385 | 633500 |
| Heptamethyl-3,3-bis(trimethylsiloxy)tetrasiloxane | 33.555 | 7121779 | 6329081 |
| 2-Monostearin | 34.053 | 2952262 | 79075 |
| Glycerol monostearate | 34.472 | 302379487 | 79075 |
| 9-Octadecenamide | 34.716 | 0 | 5283387 |
| 2,3-Dihydroxypropyl icosanoate | 36.416 | 3980275 | 3246944 |
| Cholesterol | 38.429 | 12102866 | 11025495 |
| Stigmasterol | 39.76 | 800923 | 5280794 |
| Stigmast-5-ene | 40.3 | 2708608 | 21771614 |

**Supplementary Table 5.** List of extracted metabolites using extraction solvent E (acetonitrile/water)

| **Identification** | **Retention time** | **Area under curve** | **PubChem ID** |
| --- | --- | --- | --- |
| Boric acid | 5.701 | 23321619 | 7628 |
| Lactic Acid | 7.242 | 155779670 | 107689 |
| Glycolic acid | 7.707 | 278303 | 757 |
| 1H-Azonine | 7.784 | 998300 | 13287582 |
| L-Valine | 7.854 | 1473123 | 6287 |
| Alanine | 8.159 | 2167166 | 5950 |
| Ethylene glycol | 8.205 | 3805783 | 174 |
| Glycine | 8.477 | 1615541 | 750 |
| 3-Pyridinol | 8.777 | 970561 | 7971 |
| Silane | 8.992 | 14886287 | 23953 |
| L-Leucine | 9.202 | 4401764 | 6106 |
| 4-Hydroxybutanoic acid | 9.437 | 596333 | 7302 |
| L-Proline | 9.565 | 2484408 | 145742 |
| L-Isoleucine | 9.622 | 5495186 | 6306 |
| Trisiloxane | 9.906 | 2831229 | 17860093 |
| N-Acetylaminoethanol | 10.449 | 1169597 | 14960462 |
| L-Valine | 10.574 | 833561 | 6287 |
| Benzoic Acid | 11.05 | 357662 | 243 |
| 3-Ethyl-3-methylheptadecane | 11.097 | 216819 | 11536486 |
| Diethylene glycol | 11.14 | 508831 | 8117 |
| 4-Aminobutanoic acid | 11.402 | 15822091 | 119 |
| Glycerol | 11.626 | 37655907 | 753 |
| L-Threonine | 12.269 | 2587250 | 6288 |
| 3-Aminoisobutyric acid | 12.437 | 3447730 | 64956 |
| Butanedioic acid | 12.592 | 4654901 | 1110 |
| Cyclohexasiloxane | 12.9 | 973227 | 9903713 |
| 2-Butenedioic acid | 13.056 | 2498902 | 444266 |
| Glyceric acid | 13.148 | 1261658 | 439194 |
| Myristic acid | 13.294 | 967775 | 11005 |
| Ethylphosphonic acid | 13.379 | 2825696 | 204482 |
| 6-Dimethyl(trimethylsilyl)silyloxytetradecane | 14.396 | 578909 | 590061 |
| Thiodiglycol | 14.5 | 8177157 | 5447 |
| beta-Alanine | 14.768 | 1011004 | 239 |
| L-Aspartic acid | 14.814 | 11095137 | 5960 |
| 2,4'-Bipyridine | 15.158 | 1093634 | 68488 |
| L-Hydroxyproline | 15.707 | 5809140 | 5810 |
| Succinic acid | 15.826 | 12240640 | 1110 |
| 1-Hexanol | 16.004 | 170485 | 8103 |
| Malic acid | 16.171 | 30825026 | 525 |
| Pyroglutamic acid | 16.336 | 8720110 | 7405 |
| (2-Ethoxyethoxy)acetic acid | 16.555 | 3591291 | 192850 |
| L-5-Oxoproline | 16.788 | 18654026 | 7405 |
| Acetoin | 17.079 | 2021585 | 179 |
| DL-Phenylalanine | 17.154 | 2458644 | 994 |
| 2,3,4-Trihydroxybutyric acid | 17.447 | 6242033 | 439535 |
| Pentanedioic acid | 17.854 | 1354400 | 743 |
| L-Proline | 17.979 | 1990273 | 145742 |
| Amphetamine | 18.058 | 1098751 | 3007 |
| Pimelic acid | 18.147 | 546659 | 385 |
| Triethanolamine | 18.625 | 10835385 | 7618 |
| Dodecanoic acid | 18.762 | 2405771 | 3893 |
| 2,2'-Sulfinyldiethanol | 19.174 | 952592 | 18330 |
| Propanoic acid | 19.332 | 9441883 | 1032 |
| Levoglucosan | 20.279 | 2158786 | 2724705 |
| Erythro-Pentonic acid | 20.917 | 3302572 | 90658608 |
| Nonadecane | 21.08 | 941706 | 12401 |
| Phosphoric acid | 21.284 | 18037128 | 1004 |
| Phosphorylethanolamine | 21.456 | 7396066 | 1015 |
| Citric acid | 22.214 | 6263400 | 311 |
| D-(+)-Ribono-1 | 22.395 | 62588114 | 111064 |
| Deoxyglucose | 22.481 | 26071453 | 108223 |
| Sulfurous acid | 22.686 | 503838 | 1100 |
| beta-D-Galactofuranoside | 22.755 | 469232 | 22215147 |
| D-Pinitol | 22.944 | 38510171 | 164619 |
| D-(-)-Tagatose | 23.291 | 3568082 | 92092 |
| Glucopyranose | 23.389 | 13472478 | 5793 |
| Pentadecanoic acid | 23.586 | 1278013 | 13849 |
| d-Glucose, 2,3,4,5,6-pentakis-O-(trimethylsilyl) | 23.656 | 9635809 | 522260 |
| L-Tyrosine | 23.843 | 2263508 | 6057 |
| d-Mannose, 2,3,4,5,6-pentakis-O-(trimethylsilyl) | 23.947 | 3010342 | 5378916 |
| Diisooctyl phthalate | 24.207 | 4019977 | 33934 |
| Octasiloxane | 24.346 | 4770083 | 21881078 |
| Glucopyranose | 24.856 | 16886228 | 5793 |
| Palmitic Acid | 25.072 | 306689365 | 985 |
| D-Gluconic acid | 25.199 | 4991403 | 10690 |
| 2(1H)-Pyrimidinone | 25.405 | 3972490 | 68401 |
| Scyllo-Inositol | 25.617 | 1783065 | 892 |
| Octadecane | 25.678 | 1439130 | 11635 |
| Heptadecanoic acid | 26.451 | 932062 | 10465 |
| Octadecane-1,2-diol | 26.543 | 7414257 | 89314 |
| Cyclopentanemethanol | 26.833 | 1437515 | 77195 |
| 2-Methyltetracosane | 27.071 | 1089644 | 527459 |
| Spermine | 27.32 | 1872030 | 1103 |
| Heptadecyl acetate | 27.386 | 4140022 | 69967 |
| 9,12-Octadecadienoic acid (Z,Z) | 27.462 | 20037127 | 5379173 |
| Oleic Acid | 27.577 | 7384438 | 445639 |
| Stearic acid | 27.825 | 222010162 | 5281 |
| Phosphoric acid, bis(trimethylsilyl) 2,3-bis[(trimethylsilyl)oxy]propyl ester | 27.924 | 4245721 | 553774 |
| N,N-Dimethyldodecanamide | 28.162 | 4800988 | 18159 |
| 1,1,1,5,7,7,7-Heptamethyl-3,3-bis(trimethylsiloxy)tetrasiloxane | 28.476 | 10664140 | 6329081 |
| 9,12-Octadecadienoic acid (Z,Z) | 28.65 | 1938693 | 5379173 |
| 18-Methyl-nonadecanol, trimethylsilyl ether | 29.156 | 1002285 | 91743660 |
| D-Galactofuranose, 1,2,3,5-tetrakis-O-(trimethylsilyl)-, bis(trimethylsilyl) phosphate | 29.552 | 4247381 | 553776 |
| d-Glucose, 2,3,4,5-tetrakis-O-(trimethylsilyl)-, o-methyloxime, 6-[bis(trimethylsilyl) phosphate | 29.847 | 2390988 | 90473467 |
| 1-Monomyristin | 29.97 | 14360122 | 79050 |
| Sebacic acid | 30.161 | 2221057 | 5192 |
| Octasiloxane, 1,1,3,3,5,5,7,7,9,9,11,11,13,13,15,15-hexadecamethyl | 30.286 | 5396325 | 6329087 |
| Eicosanoic acid | 30.326 | 6318356 | 10467 |
| Phenol, 2,2'-methylenebis[6-(1,1-dimethylethyl)-4-methyl | 30.641 | 1520808 | 8398 |
| N,N-Dimethyldodecanamide | 30.802 | 2948404 | 18159 |
| Myo-Inositol, 1,3,4,5,6-pentakis-O-(trimethylsilyl)-, bis(trimethylsilyl) phosphate | 30.869 | 7284716 | 553143 |
| 2-Methyl-6-tert-butylphenol | 31.107 | 56096509 | 16678 |
| benzene, 1,1'-methylenebis[3-(1,1-dimethylethyl)-5-methyl-4-[(trimethylsilyl)oxy] | 31.875 | 42673697 | 91697106 |
| Heptamethyl-3,3-bis(trimethylsiloxy)tetrasiloxane | 31.982 | 9586660 | 6329081 |
| 1-Monopalmitin | 32.358 | 725688268 | 3084463 |
| 5-Methyluridine | 33.045 | 2665893 | 445408 |
| Adenosine | 33.269 | 10346826 | 60961 |
| Heptadecanoic acid, glycerine-(1)-monoester, bis-O-trimethylsilyl | 33.364 | 1889228 | 633500 |
| Heptamethyl-3,3-bis(trimethylsiloxy)tetrasiloxane | 33.555 | 6856043 | 6329081 |
| 2-Monostearin | 34.053 | 1864294 | 79075 |
| Glycerol monostearate | 34.472 | 355277040 | 79075 |
| 9-Octadecenamide | 34.716 | 53146417 | 5283387 |
| 2,3-Dihydroxypropyl icosanoate | 36.416 | 3565644 | 3246944 |
| Cholesterol | 38.429 | 8293218 | 11025495 |
| Stigmasterol | 39.76 | 674722 | 5280794 |
| Stigmast-5-ene | 40.3 | 2036593 | 21771614 |

**Supplementary Table 6.** Main metabolic pathways involving metabolites preferentially extracted using the organic based solvents NH_4_OH and EA

| **Metabolite Set** | **total** | **expected** | **hits** | **p-value** |
| --- | --- | --- | --- | --- |
| Glycerolipid Metabolism | 25 | 0.537 | 3 | 0.0146 |
| Warburg Effect | 58 | 1.25 | 4 | 0.0315 |
| Gluconeogenesis | 35 | 0.752 | 3 | 0.0361 |
| Lactose Synthesis | 20 | 0.43 | 2 | 0.0663 |
| Glutathione Metabolism | 21 | 0.451 | 2 | 0.0723 |
| Glycolysis | 25 | 0.537 | 2 | 0.0981 |
| Valine, Leucine and Isoleucine Degradation | 60 | 1.29 | 3 | 0.133 |
| Fatty Acid Biosynthesis | 35 | 0.752 | 2 | 0.171 |
| Lactose Degradation | 9 | 0.193 | 1 | 0.178 |
| Galactose Metabolism | 38 | 0.816 | 2 | 0.195 |
| Glycerol Phosphate Shuttle | 11 | 0.236 | 1 | 0.213 |
| Glucose-Alanine Cycle | 13 | 0.279 | 1 | 0.247 |
| Ketone Body Metabolism | 13 | 0.279 | 1 | 0.247 |
| Thyroid hormone synthesis | 13 | 0.279 | 1 | 0.247 |
| Pyruvate Metabolism | 48 | 1.03 | 2 | 0.276 |
| Steroid Biosynthesis | 48 | 1.03 | 2 | 0.276 |
| Glutamate Metabolism | 49 | 1.05 | 2 | 0.284 |
| Beta Oxidation of Very Long Chain Fatty Acids | 17 | 0.365 | 1 | 0.311 |
| Arginine and Proline Metabolism | 53 | 1.14 | 2 | 0.317 |
| Butyrate Metabolism | 19 | 0.408 | 1 | 0.341 |
| Mitochondrial Electron Transport Chain | 19 | 0.408 | 1 | 0.341 |
| Catecholamine Biosynthesis | 20 | 0.43 | 1 | 0.355 |
| Vitamin B6 Metabolism | 20 | 0.43 | 1 | 0.355 |
| Pyrimidine Metabolism | 59 | 1.27 | 2 | 0.365 |
| Carnitine Synthesis | 22 | 0.473 | 1 | 0.383 |
| Transfer of Acetyl Groups into Mitochondria | 22 | 0.473 | 1 | 0.383 |
| Bile Acid Biosynthesis | 65 | 1.4 | 2 | 0.413 |
| Cysteine Metabolism | 26 | 0.559 | 1 | 0.435 |
| Inositol Phosphate Metabolism | 26 | 0.559 | 1 | 0.435 |
| Oxidation of Branched Chain Fatty Acids | 26 | 0.559 | 1 | 0.435 |
| Phytanic Acid Peroxisomal Oxidation | 26 | 0.559 | 1 | 0.435 |
| Plasmalogen Synthesis | 26 | 0.559 | 1 | 0.435 |
| Mitochondrial Beta-Oxidation of Medium Chain Saturated Fatty Acids | 27 | 0.58 | 1 | 0.448 |
| Mitochondrial Beta-Oxidation of Long Chain Saturated Fatty Acids | 28 | 0.602 | 1 | 0.46 |
| Phenylalanine and Tyrosine Metabolism | 28 | 0.602 | 1 | 0.46 |
| Selenoamino Acid Metabolism | 28 | 0.602 | 1 | 0.46 |
| Urea Cycle | 29 | 0.623 | 1 | 0.472 |
| Ammonia Recycling | 32 | 0.688 | 1 | 0.506 |
| Citric Acid Cycle | 32 | 0.688 | 1 | 0.506 |
| Fructose and Mannose Degradation | 32 | 0.688 | 1 | 0.506 |
| Inositol Metabolism | 33 | 0.709 | 1 | 0.517 |
| Fatty Acid Elongation In Mitochondria | 35 | 0.752 | 1 | 0.538 |
| Nicotinate and Nicotinamide Metabolism | 37 | 0.795 | 1 | 0.559 |
| Sphingolipid Metabolism | 40 | 0.859 | 1 | 0.588 |
| Propanoate Metabolism | 42 | 0.902 | 1 | 0.606 |
| Fatty acid Metabolism | 43 | 0.924 | 1 | 0.615 |
| Steroidogenesis | 43 | 0.924 | 1 | 0.615 |
| Glycine and Serine Metabolism | 59 | 1.27 | 1 | 0.733 |
| Tyrosine Metabolism | 72 | 1.55 | 1 | 0.802 |
| Purine Metabolism | 74 | 1.59 | 1 | 0.811 |

**Supplementary Table 7.** Main metabolic pathways involving metabolites preferentially extracted using the organic based solvents FA, MeOH.H2O and MeCN.H2O

| **Metabolite Set** | **total** | **expected** | **hits** | **p-value** |
| --- | --- | --- | --- | --- |
| Glutathione Metabolism | 21 | 1.27 | 4 | 0.0335 |
| Glutamate Metabolism | 49 | 2.97 | 6 | 0.0694 |
| Malate-Aspartate Shuttle | 10 | 0.605 | 2 | 0.119 |
| Gluconeogenesis | 35 | 2.12 | 4 | 0.156 |
| Glucose-Alanine Cycle | 13 | 0.787 | 2 | 0.183 |
| Glycerolipid Metabolism | 25 | 1.51 | 3 | 0.189 |
| Arginine and Proline Metabolism | 53 | 3.21 | 5 | 0.212 |
| Selenoamino Acid Metabolism | 28 | 1.7 | 3 | 0.237 |
| Propanoate Metabolism | 42 | 2.54 | 4 | 0.246 |
| Urea Cycle | 29 | 1.76 | 3 | 0.254 |
| Warburg Effect | 58 | 3.51 | 5 | 0.271 |
| Alanine Metabolism | 17 | 1.03 | 2 | 0.275 |
| Ammonia Recycling | 32 | 1.94 | 3 | 0.305 |
| Lactose Synthesis | 20 | 1.21 | 2 | 0.344 |
| Fatty Acid Biosynthesis | 35 | 2.12 | 3 | 0.357 |
| Carnitine Synthesis | 22 | 1.33 | 2 | 0.389 |
| Transfer of Acetyl Groups into Mitochondria | 22 | 1.33 | 2 | 0.389 |
| Lactose Degradation | 9 | 0.545 | 1 | 0.431 |
| Glycolysis | 25 | 1.51 | 2 | 0.454 |
| Glycine and Serine Metabolism | 59 | 3.57 | 4 | 0.486 |
| Glycerol Phosphate Shuttle | 11 | 0.666 | 1 | 0.499 |
| Valine, Leucine and Isoleucine Degradation | 60 | 3.63 | 4 | 0.5 |
| Phosphatidylethanolamine Biosynthesis | 12 | 0.727 | 1 | 0.529 |
| Ketone Body Metabolism | 13 | 0.787 | 1 | 0.558 |
| Thyroid hormone synthesis | 13 | 0.787 | 1 | 0.558 |
| Phosphatidylcholine Biosynthesis | 14 | 0.848 | 1 | 0.585 |
| Vitamin K Metabolism | 14 | 0.848 | 1 | 0.585 |
| Citric Acid Cycle | 32 | 1.94 | 2 | 0.589 |
| Beta-Alanine Metabolism | 34 | 2.06 | 2 | 0.623 |
| Aspartate Metabolism | 35 | 2.12 | 2 | 0.639 |
| Beta Oxidation of Very Long Chain Fatty Acids | 17 | 1.03 | 1 | 0.657 |
| Purine Metabolism | 74 | 4.48 | 4 | 0.672 |
| Spermidine and Spermine Biosynthesis | 18 | 1.09 | 1 | 0.678 |
| Galactose Metabolism | 38 | 2.3 | 2 | 0.684 |
| Butyrate Metabolism | 19 | 1.15 | 1 | 0.698 |
| Mitochondrial Electron Transport Chain | 19 | 1.15 | 1 | 0.698 |
| Pyrimidine Metabolism | 59 | 3.57 | 3 | 0.709 |
| Sphingolipid Metabolism | 40 | 2.42 | 2 | 0.712 |
| Catecholamine Biosynthesis | 20 | 1.21 | 1 | 0.717 |
| Threonine and 2-Oxobutanoate Degradation | 20 | 1.21 | 1 | 0.717 |
| Vitamin B6 Metabolism | 20 | 1.21 | 1 | 0.717 |
| Betaine Metabolism | 21 | 1.27 | 1 | 0.734 |
| Methionine Metabolism | 43 | 2.6 | 2 | 0.749 |
| Bile Acid Biosynthesis | 65 | 3.94 | 3 | 0.771 |
| Pyruvate Metabolism | 48 | 2.91 | 2 | 0.803 |
| Steroid Biosynthesis | 48 | 2.91 | 2 | 0.803 |
| Cysteine Metabolism | 26 | 1.57 | 1 | 0.807 |
| Inositol Phosphate Metabolism | 26 | 1.57 | 1 | 0.807 |
| Oxidation of Branched Chain Fatty Acids | 26 | 1.57 | 1 | 0.807 |
| Phytanic Acid Peroxisomal Oxidation | 26 | 1.57 | 1 | 0.807 |
| Plasmalogen Synthesis | 26 | 1.57 | 1 | 0.807 |
| Mitochondrial Beta-Oxidation of Medium Chain Saturated Fatty Acids | 27 | 1.63 | 1 | 0.819 |
| Mitochondrial Beta-Oxidation of Long Chain Saturated Fatty Acids | 28 | 1.7 | 1 | 0.83 |
| Phenylalanine and Tyrosine Metabolism | 28 | 1.7 | 1 | 0.83 |
| Fructose and Mannose Degradation | 32 | 1.94 | 1 | 0.869 |
| Inositol Metabolism | 33 | 2 | 1 | 0.877 |
| Fatty Acid Elongation In Mitochondria | 35 | 2.12 | 1 | 0.892 |
| Nicotinate and Nicotinamide Metabolism | 37 | 2.24 | 1 | 0.905 |
| Porphyrin Metabolism | 40 | 2.42 | 1 | 0.922 |
| Fatty acid Metabolism | 43 | 2.6 | 1 | 0.936 |
| Histidine Metabolism | 43 | 2.6 | 1 | 0.936 |
| Steroidogenesis | 43 | 2.6 | 1 | 0.936 |
| Tyrosine Metabolism | 72 | 4.36 | 2 | 0.943 |
| Tryptophan Metabolism | 60 | 3.63 | 1 | 0.979 |

**Supplementary Table 8.** List of metabolites used with their corresponding number shown in unsupervised hierarchical clustering

| 1 | Boric acid, 3TMS derivative |
| --- | --- |
| 2 | Lactic Acid, 2TMS derivative |
| 3 | Glycolic acid, 2TMS derivative |
| 4 | 1H-Azonine, octahydro-1-nitroso- |
| 5 | L-Valine, TMS derivative |
| 6 | Alanine, 2TMS derivative |
| 7 | Ethylene glycol, 2TMS derivative |
| 8 | Glycine, 2TMS derivative |
| 9 | 3-Pyridinol, TMS derivative |
| 10 | Silane, [(1-methoxy-1,3-propanediyl)bis(oxy)]bis[trimethyl- |
| 11 | L-Leucine, TMS derivative |
| 12 | 4-Hydroxybutanoic acid, 2TMS derivative |
| 13 | L-Proline, TMS derivative |
| 14 | L-Isoleucine, TMS derivative |
| 15 | Trisiloxane |
| 16 | N-Acetylaminoethanol, TMS derivative |
| 17 | L-Valine, 2TMS derivative |
| 18 | Benzoic Acid, TMS derivative |
| 19 | 3-Ethyl-3-methylheptadecane |
| 20 | Diethylene glycol, 2TMS derivative |
| 21 | 4-Aminobutanoic acid, 3TMS derivative |
| 22 | Glycerol, 3TMS derivative |
| 23 | L-Threonine, 2TMS derivative |
| 24 | 3-Aminoisobutyric acid, 3TMS derivative |
| 25 | Butanedioic acid, 2TMS derivative |
| 26 | Cyclohexasiloxane, dodecamethyl- |
| 27 | 2-Butenedioic acid, (E)-, 2TMS derivative |
| 28 | Glyceric acid, 3TMS derivative |
| 29 | Myristic acid, TMS derivative |
| 30 | Ethylphosphonic acid, 2TMS derivative |
| 31 | 6-Dimethyl(trimethylsilyl)silyloxytetradecane |
| 32 | Thiodiglycol, 2TMS derivative |
| 33 | .beta.-Alanine, 3TMS derivative |
| 34 | L-Aspartic acid, 2TMS derivative |
| 35 | 2,4'-Bipyridine |
| 36 | L-Hydroxyproline, (E)-, 2TMS derivative |
| 37 | Succinic acid, 3,5-difluorophenyl 2-(dimethylamino)ethyl ester |
| 38 | 1-Hexanol, 5-methyl-2-(1-methylethyl)- |
| 39 | Malic acid, 3TMS derivative |
| 40 | Pyroglutamic acid, TMS derivative |
| 41 | (2-Ethoxyethoxy)acetic acid, TMS derivative |
| 42 | L-5-Oxoproline, , 2TMS derivative |
| 43 | Pyroglutamic acid, TMS derivative |
| 44 | Acetoin, TMS derivative |
| 45 | DL-Phenylalanine, TMS derivative |
| 46 | 2,3,4-Trihydroxybutyric acid tetrakis(trimethylsilyl) deriv |
| 47 | Pentanedioic acid, 2-[(trimethylsilyl)oxy]-, bis(trimethylsilyl) ester |
| 48 | L-Proline, 2TMS derivative |
| 49 | Amphetamine, 2TMS derivative |
| 50 | Pimelic acid, 2TMS derivative |
| 51 | Triethanolamine, 3TMS derivative |
| 52 | Dodecanoic acid, TMS derivative |
| 53 | 2,2'-Sulfinyldiethanol, 2TMS derivative |
| 54 | Propanoic acid, 3-[methyl[2-oxo-2-[(trimethylsilyl)oxy]ethyl]amino]-3-oxo-, trimethylsilyl ester |
| 55 | Levoglucosan, 3TMS derivative |
| 56 | Erythro-Pentonic acid, 2-deoxy-3,4,5-tris-O-(trimethylsilyl)-, trimethylsilyl ester |
| 57 | Nonadecane |
| 58 | Phosphoric acid, bis(trimethylsilyl) 2,3-bis[(trimethylsilyl)oxy]propyl ester |
| 59 | Phosphorylethanolamine, 4TMS derivative |
| 60 | Myristic acid, TMS derivative |
| 61 | Citric acid, 4TMS derivative |
| 62 | D-(+)-Ribono-1,4-lactone, 3TMS derivative |
| 63 | Deoxyglucose, 4TMS derivative |
| 64 | Sulfurous acid, decyl 2-propyl ester |
| 65 | .beta.-D-Galactofuranoside, ethyl 2,3,5,6-tetrakis-O-(trimethylsilyl)- |
| 66 | D-Pinitol, pentakis(trimethylsilyl) ether |
| 67 | D-(-)-Tagatose, pentakis(trimethylsilyl) ether, methyloxime (anti) |
| 68 | Glucopyranose, 5TMS derivative |
| 69 | Pentadecanoic acid, TMS derivative |
| 70 | d-Glucose, 2,3,4,5,6-pentakis-O-(trimethylsilyl)-, o-methyloxyme, (1E)- |
| 71 | L-Tyrosine, 3TMS derivative |
| 72 | d-Mannose, 2,3,4,5,6-pentakis-O-(trimethylsilyl)-, o-methyloxyme, (1E)- |
| 73 | Diisooctyl phthalate |
| 74 | Octasiloxane |
| 75 | Myristic acid, TMS derivative |
| 76 | Glucopyranose, 5TMS derivative |
| 77 | Palmitic Acid, TMS derivative |
| 78 | D-Gluconic acid, 6TMS derivative |
| 79 | 2(1H)-Pyrimidinone, 1-[2,3-bis-O-(trimethylsilyl)-.beta |
| 80 | Scyllo-Inositol, 6TMS derivative |
| 81 | Octadecane, 5-methyl- |
| 82 | Scyllo-Inositol, 6TMS derivative |
| 83 | Heptadecanoic acid, TMS derivative |
| 84 | Octadecane-1,2-diol, 2TMS derivative |
| 85 | Cyclopentanemethanol, .alpha.-cyclohexyl-2-nitro- |
| 86 | 2-Methyltetracosane |
| 87 | Spermine, 6TMS derivative |
| 88 | Heptadecyl acetate |
| 89 | 9,12-Octadecadienoic acid (Z,Z)-, TMS derivative |
| 90 | Oleic Acid, (Z)-, TMS derivative |
| 91 | Stearic acid, TMS derivative |
| 92 | Phosphoric acid, bis(trimethylsilyl) 2,3-bis[(trimethylsilyl)oxy]propyl ester |
| 93 | N,N-Dimethyldodecanamide |
| 94 | 1,1,1,5,7,7,7-Heptamethyl-3,3-bis(trimethylsiloxy)tetrasiloxane |
| 95 | 9,12-Octadecadienoic acid (Z,Z)-, TMS derivative |
| 96 | 18-Methyl-nonadecanol, trimethylsilyl ether |
| 97 | D-Galactofuranose, 1,2,3,5-tetrakis-O-(trimethylsilyl)-, bis(trimethylsilyl) phosphate |
| 98 | d-Glucose, 2,3,4,5-tetrakis-O-(trimethylsilyl)-, o-methyloxime, 6-[bis(trimethylsilyl) phosphate] |
| 99 | 1-Monomyristin, 2TMS derivative |
| 100 | Sebacic acid, 2TMS derivative |
| 101 | Octasiloxane, 1,1,3,3,5,5,7,7,9,9,11,11,13,13,15,15-hexadecamethyl- |
| 102 | Eicosanoic acid, TMS |
| 103 | Phenol, 2,2'-methylenebis[6-(1,1-dimethylethyl)-4-methyl- |
| 104 | N,N-Dimethyldodecanamide |
| 105 | Myo-Inositol, 1,3,4,5,6-pentakis-O-(trimethylsilyl)-, bis(trimethylsilyl) phosphate |
| 106 | 2-Methyl-6-tert-butylphenol, TMS derivative |
| 107 | benzene, 1,1'-methylenebis[3-(1,1-dimethylethyl)-5-methyl-4-[(trimethylsilyl)oxy]- |
| 108 | 1,1,1,5,7,7,7-Heptamethyl-3,3-bis(trimethylsiloxy)tetrasiloxane |
| 109 | 1-Monopalmitin, 2TMS derivative |
| 110 | 5-Methyluridine, 3TMS derivative |
| 111 | Adenosine, 4TMS derivative |
| 112 | Heptadecanoic acid, glycerine-(1)-monoester, bis-O-trimethylsilyl- |
| 113 | 1,1,1,5,7,7,7-Heptamethyl-3,3-bis(trimethylsiloxy)tetrasiloxane |
| 114 | 2-Monostearin, 2TMS derivative |
| 115 | Glycerol monostearate, 2TMS derivative |
| 116 | 9-Octadecenamide, (Z)- |
| 117 | 2,3-Dihydroxypropyl icosanoate, 2TMS derivative |
| 118 | Cholesterol, TMS derivative |
| 119 | Stigmasterol, TMS derivative |
| 120 | Stigmast-5-ene, 3.beta.-(trimethylsiloxy)-, (24S)- |


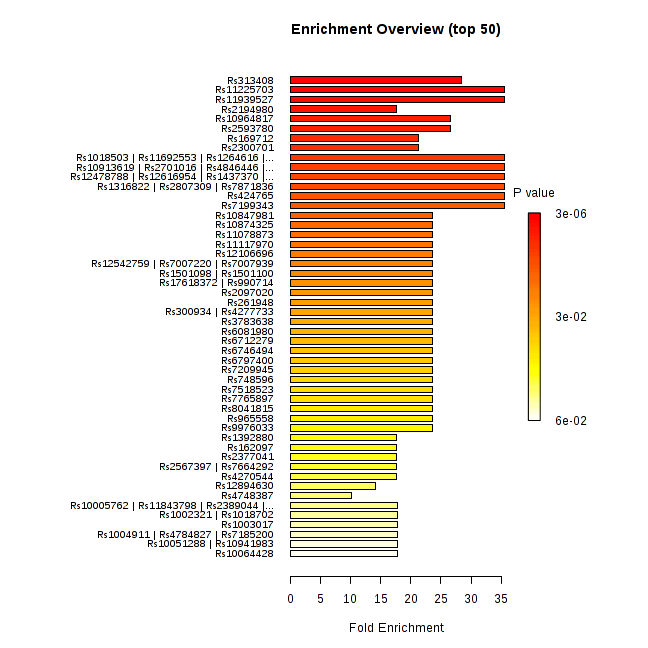


**Supplementary Figure 1.** Summary plot for Metabolite Set Enrichment Analysis (MSEA) of SNPs that map to metabolites


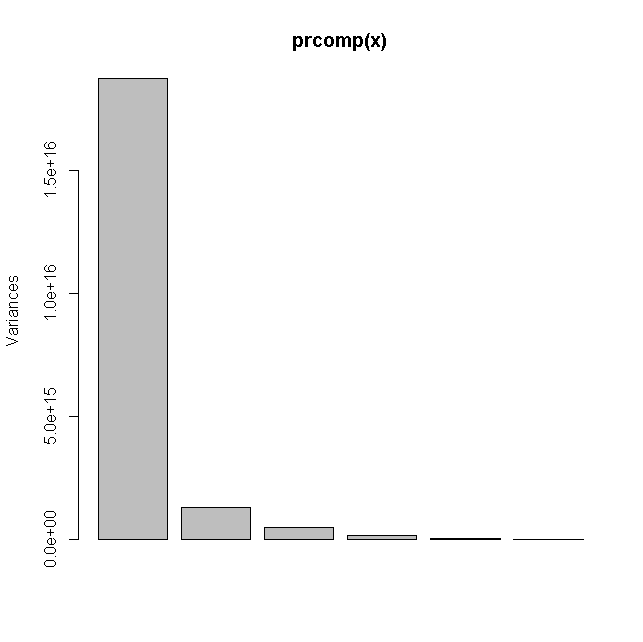


**Supplementary Figure 2. Principle component analysis of the metabolic data generate from GC-MS**
